# Limits and possibilities for the development of public health research in the legal system

**DOI:** 10.11606/s1518-8787.2022056004203

**Published:** 2022-08-16

**Authors:** Nayla Rochele Nogueira de Andrade, Carlos Francisco Oliveira Nunes, Felipe Braga Albuquerque, Carmem E. Leitão Araújo, Anderson Fuentes Ferreira, Adriana da Silva dos Reis, Alberto Novaes Ramos

**Affiliations:** I Universidade Federal do Ceará Faculdade de Medicina Programa de Pós-Graduação em Saúde Pública Fortaleza CE Brasil Universidade Federal do Ceará. Faculdade de Medicina. Programa de Pós-Graduação em Saúde Pública. Fortaleza, CE, Brasil; II Universidade Federal de Santa Catarina Programa de Pós-Graduação em Engenharia e Gestão do Conhecimento Florianópolis Santa Catarina Brasil Universidade Federal de Santa Catarina. Programa de Pós-Graduação em Engenharia e Gestão do Conhecimento. Florianópolis, Santa Catarina, Brasil; III Universidade Federal do Ceará Faculdade de Direito Departamento de Direito Público Fortaleza CE Brasil Universidade Federal do Ceará. Faculdade de Direito. Departamento de Direito Público. Fortaleza, CE, Brasil; IV Universidade Federal do Ceará Faculdade de Direito Programa de Pós-Graduação em Direito Fortaleza CE Brasil Universidade Federal do Ceará. Faculdade de Direito. Programa de Pós-Graduação em Direito. Fortaleza, CE, Brasil; V Universidade Federal do Ceará Faculdade de Medicina Departamento de Saúde Comunitária Fortaleza CE Brasil Universidade Federal do Ceará. Faculdade de Medicina. Departamento de Saúde Comunitária. Fortaleza, CE, Brasil; VI NHR Brasil Fortaleza CE Brasil Netherlands Hanseniasis Relief Brasil - NHR Brasil. Fortaleza, CE, Brasil

**Keywords:** Judicial Decisions, Jurisprudence, Resources for Research, Health Law, Public Health

## Abstract

**OBJECTIVE:**

To characterize databases of the courts of justice of Brazil as a potential tool for research in Collective Health, in its interface with the legal sciences.

**METHODS:**

Cross-sectional study of quantitative and descriptive nature, focusing on analysis of strategic management and judicial systems.

**RESULTS:**

Databases used by the Common Justice in the Federation Units to systematize judicial processes were identified and analyzed. A total of 123 databases were found in the courts of justice per state, with emphasis on the South and Northeast regions, in contrast to the North region, which has a smaller number of systems. This large number of judicial systems limits access to legal operators, and hinders the collection of evidence by health researchers and, consequently, impacts the strategic management of the Executive Branch. There were limitations from design to transparent and democratic data extraction by the users themselves, as well as restricted integration between bases.

**CONCLUSIONS:**

Although advances have been made in recent years by the courts of justice to unify these databases, the multiplicity of information systems used in the Common State Justice complicates the management of knowledge, limits the development of research, even when carried out by lawyers or researchers in the legal area, as well as generates slow data extraction for public management. It is recognized the need for additional efforts for standardization, as well as for improvement of these databases, expanding access, transparency and integration with a view to a transdisciplinary look between the field of Law and Collective Health.

## INTRODUCTION

Paths to reflection and research on potential conflicts between the political and legal systems in public health management necessarily include the recognition of aspects involving the distribution and allocation of scarce resources in society^1^. Particularly in a country with the territorial dimension of Brazil, with serious social inequalities that impact on distinct and transitional epidemiological patterns, it becomes even more complex to determine priorities in the health system^2^.

The number of lawsuits in the Judiciary requiring health goods and services has grown significantly in Brazil, especially after 2007, giving rise to the phenomenon of judicialization of the right to public health, which in this work is synonymous with lawsuits against a state entity, demanding health goods and/or services^3^.

The greater involvement of the Judiciary regarding health policies makes up a greater discussion on the “*judicialization of politics*”, an expression equivalent to the “*politicization of justice*”, a reflection of its expansion in the decision-making process of contemporary democracies. In this sense, the judicialization of the right to health emerges as a conflict between the institutionalized system of political action and the legal system^4^.

The judicialization of the right to health has generated increasingly frequent debates in view of its multiple uses and meanings^5^, on the one hand, it reinforces the legal dimension of citizenship, materializing a right guaranteed in the Federal Constitution of 1988, which in its Article 196 affirms that health is a right of all and a duty of the State^6^; on the other, it can reinforce conflicts in the federative governance of the Unified Health System (*Sistema Único de Saúde -* SUS), limiting the ability of the executive power to plan, implement and monitor health policies through rational and equitable criteria^7^. The individual character of interventions and the privilege of people with greater knowledge, financial resources or other conditions of differentiated access to justice are also pointed out as critical problems^8^.

Certainly, one of the effects of this judicialization phenomenon is the expansion of the interfaces between research in the fields of health and legal sciences. There is an increase in the number of studies and articles published on the subject, with a view to dimensioning and greater understanding of this phenomenon^3,9^. For example, there are studies with analysis of databases of the Judiciary in the state of Rio de Janeiro on the judicialization of health^10,11^, in the state of Ceará on the phenomenon with recognition of the limits for analysis in the light of epidemiology^3^, and the Federal District with the analysis of the reality of judicialization in health^12^. In a broader approach to the country, the National Council of Justice (*Conselho Nacional de Justiça* - CNJ) sought to characterize the phenomenon and bring additional reflections on the subject^1,13^.

As the phenomenon of judicialization grows, there is the weakness of the Judiciary’s information systems in the systematization of data and public access systems to several courts with the potential to access and use information systems. As a result, it may make it impossible or limit fundamental analyses for the implementation of public policies based on scientific evidence, due to limitations and inconsistencies between the databases, in addition to underestimating the processes of judicialization of health^1,9,11,13-15^.

The criticisms and limitations indicate the need to rethink and better understand the different databases, many of which do not have access to researchers^3,11^. This study aims to characterize the databases of the Courts of Justice (*Tribunal de Justiça* - TJ) of Brazil as a potential tool for research in Collective Health in its interfaces with the legal sciences. The recognition of the quantity and quality of information available is strategic for more consistent and purposeful analyses, which includes better delimitation of the phenomenon of judicialization of public health policy in Brazil.

## METHODS

### Study Design

Descriptive cross-sectional study of national scope based on data collected from the State Courts of Justice of the country. The data collection process was carried out between 2019 and 2021 with the identification and characterization of physical and virtual databases used to systematize judicial processes and to recognize the process of filing actions related to public health.

The study was carried out in two stages, both based on formal data and information requirements from the TJ’s ombudsmen via filling out electronic forms available to each one, a process complemented by systematic consultation of official websites. All manifestations followed the rules available in the electronic addresses and internal guidelines directed by each court.

The first stage with the ombudsmen was based on demand composition via e-mail, telephone contact, and specific electronic form available on the websites of the TJ, following specific internal protocols in each Federation Unit (*Unidade da Federação* - UF). The follow-up of the request was performed with the protocol number generated at the time of the request.

The TJs should respond to three specific items:

- What are the names and dates of implementation of the systems used by the operators of law of this UF to register and monitor the physical judicial proceedings filed?
- What are the names and dates of implementation of the systems used by the law operators of this UF to register and monitor the filing of virtual lawsuits?
- Any observation that the TJ deems pertinent.

After completing the first stage, one decided to complement the data obtained from a second stage, which consisted of sending new requests to the TJ for further detailing of the process of protocol of actions that deal with public health from three questions:

- In the available information systems, referred to by the court in the first stage, what is the branch, in the subject tree, to classify and file an action that deals with health? Is there a difference in the protocol when it comes to public health and private health?
- Is it possible to carry out the protocol of a process in the competence of the public treasury that is in the health area, without the registration being identified that is in the health area, for example: register as “administrative act/annulment” – but be related to the supply of medicines?
- Does the distribution sector make adequacy/compatibility in the registration of actions, correcting any petition errors by the professionals who make the registrations?

### Data Analysis

For the first stage, in possession of the collected information, the data were consolidated and organized into tables, with preliminary descriptive analysis. In the few cases in which the same database was informed by the TJ as containing physical and virtual processes, this data was computed only once, to avoid overestimating the local reality.

For the second stage on the process of protocol of actions that deal with public health, the three questions answered by the TJ were consolidated and analyzed descriptively.

### Ethical Aspects

The data in this study are secondary and public, according to the principle of advertising of Art. 5th, item LX, of the Federal Constitution of 1988, Art. 189 of the Code of Civil Procedure, and Law No. 12,527/11^11^, and other provisions.

In addition, the project was submitted to the Research Ethics Committee of the *Universidade Federal do Ceará* CEP/UFC/PROPESQ on the Brazil Platform, which had as its opinion a statement stating that “*the project does not apply to the evaluation of the Research Ethics Committee, since it is a research that uses freely accessible information and because it uses a database, whose information is aggregated, without the possibility of individual identification, in a manner similar to the provisions of CNS Resolution No. 510, of April 7, 2016*”.

## RESULTS

### Step 1

In the state of Ceará (CE), data were collected *in loco*, due to the ease of access of the research team.

The TJ of Acre (AC), Amapá (AP), Amazonas (AM), Pará (PA), Rondônia (RO), Roraima (RR), Alagoas (AL), Bahia (BA), Maranhão (MA), Rio Grande do Norte (RN), Distrito Federal (DF), Mato Grosso (MT), Espírito Santo (ES), Minas Gerais (MG), São Paulo (SP) and Rio Grande do Sul (RS) returned contacts via email, corresponding to 59.2% of the total.

For the TJ of Tocantins (TO), Sergipe (SE), Paraíba (PB), Pernambuco (PE), Piauí (PI), Goiás (GO), Mato Grosso do Sul (MS), Rio de Janeiro (RJ), Paraná (PR) and Santa Catarina (SC), direct telephone contacts were made to the ombudsmen, as well as to the sectors responsible for the information technology service of the courts. In addition, systematic searches were carried out on websites. In all cases, it was possible to compose perspectives for the three items of interest at this stage.

The result of the first stage of the study is summarized in Tables 1 and 2. Table 1 characterizes the existing databases of information systems in each TJ of the UFs of the country, as well as those that are intended for the registration of physical processes (old databases) and the registration of virtual processes (modern databases).

**Table 1:** Number and specification by federation unit and region of Brazil of the databases in State Courts of Justice, 2021.

Federative Units (UF)	Databases	Physical processes	Databases	Virtual Processes	Overall Total	Total without duplication
	Abbreviation	n (%)	Abbreviation	n (%)	n (%)	n (%)
North		9 (20.5)		23 (23.7)	32 (33.0)	26 (26.8)
Acre (AC)	No data	0 (0)	e-SAJPG/e-SAJSG/SEEU	3 (13.0)	3 (9.4)	3 (11.5)
Amapá (AP)	Tucujuris	1 (11.1)	Tucujurisweb/SEEU/PJE1G	3 (13.0)	4 (12.5)	3 (11.5)
Amazonas (AM)	e-SAJ PG/e-SAJ SG/Projudi	3 (33.3)	e-SAJ PG/e-SAJ SG/Projudi	3 (13.0)	6 (18.8)	3 (11.5)
Pará (PA)	Libra	1 (11.1)	PJE1G/PJE2G/SEEU/Projudi	4 (17.4)	5 (15.6)	5 (19.2)
Rondônia (RO)	PJE1G, PJE2G	2 (22.2)	PJE1G, PJE2G	2 (8.7)	4 (12.5)	2 (7.7)
Roraima (RR)	Siscom	1 (11.1)	Projudi, SEEU, PJE1G, PJE2G	4 (17.4)	5 (15.6)	5 (19.2)
Tocantins (TO)	Sicap	1 (11.1)	EPROC1G/EPRO2G/Projudi/SPROC	4 (17.4)	5 (15.6)	5 (19.2)
Northeast		15 (34.1)		32 (33.0)	47 (48.5)	44 (45.4)
Alagoas (AL)	e-SAJ PG/SAJ SG	2 (13.3)	e-SAJ PG/e-SAJ SG	2 (6.3)	4 (8.5)	2 (4.5)
Bahia (BA)	Saipro	1 (6.7)	e-SAJ PG/e-SAJ SG/PJE1G/Projudi	4 (12.5)	5 (10.6)	5 (11.4)
Ceará (CE)	Projudi/SPROC	2 (13.3)	e-SAJPG/e-SAJSG, PJE1, PJE2	4 (12.5)	6 (12.8)	6 (13.6)
Maranhão (MA)	THEMIS1G/THEMIS2G	2 (13.3)	Projudi/VEP/PJE1G/PJE2/SEEU	5 (15.6)	7 (14.9)	7 (15.9)
Paraíba (PB)	E-JUS/VEP	2 (13.3)	PJE1G/PJE2G/Consulta Unificada Beta	3 (9.4)	5 (10.6)	5 (11.4)
Pernambuco (PE)	JUDWIN	1 (6.7)	PJE1G, PJE2G, Projudi, SEEU	4 (12.5)	5 (10.6)	5 (11.4)
Piauí (PI)	Themisweb/Themiswebrecursal	2 (13.3)	Projudi/Projudirecursal/SEEU/e-TJPI PJ1G/PJE2G	6 (18.8)	8 (17.0)	8 (18.2)
Rio Grande do Norte (RN)	e-SAJ PG5, e-SAJ SG3	2 (13.3)	e-SAJ PG Digital, PJE1G, PJE2G	3 (9.4)	5 (10.6)	5 (11.4)
Sergipe (SE)	SCPV	1 (6.7)	SCPV	1 (3.1)	2 (4.3)	1 (2.3)
Midwest		7 (15.9)		12 (12.4)	19 (19.6)	16 (16.5)
Federal District (DF)	QVT/SISTJ Gráfico/SISTJWEB	3 (42.9)	Projudi/PJE1/PJE2/SEEU	4 (33.3)	7 (36.8)	7 (43.8)
Goiás (GO)	Physical proccess consultation portal 1st and 2nd degree	1 (14.3)	Projudi/SEEU	2 (16.7)	3 (15.8)	3 (18.8)
Mato Grosso do Sul (MS)	e- SAJPG/e-SAJSG	2 (28.6)	e-SAJPG/e-SAJSG	2 (16.7)	4 (21.1)	2 (12.5)
Mato Grosso (MT)	Sistema Apolo	1 (14.3)	Apolo Eletrônico/PJE1G/PJE2G Projudi	4 (33.3)	5 (26.3)	4 (25.0)
Southeast		7 (15.9)		13 (13.4)	20 (20.6)	19 (19.6)
Espírito Santo (ES)	Sistema de Segunda Instância EJUD/SIEP	3 (42.9)	Projudi/PJE1G/SEEU	3 (23.1)	6 (30.0)	6 (31.6)
Minas Gerais (MG)	SIAP/Siscom	2 (28.6)	PJE1G/PJE2G/Projudi/SEEU	4 (30.8)	6 (30.0)	6 (31.6)
Rio de Janeiro (RJ)	PJERJ	1 (14,3)	E-mail, Projudi, PJE1G, PJERJ	4 (30.8)	5 (25.0)	4 (21.1)
São Paulo (SP)	VEC	1 (14,3)	e-SAJPG/e-SAJSG	2 (15.4)	3 (15.0)	3 (15.8)
South		6 (13,6)		17 (17.5)	23 (23.7)	18 (18.6)
Paraná (PR)	Portal TJPR Varas Estatizadas com Processos Físicos (em papel)	1 (16,7)	Projudi/1GCÍVEL/1GVEP 1GCRIMINAL/2G	5 (29.4)	6 (26.1)	6 (33.3)
Rio Grande do Sul (RS)	THEMIS1G/THEMIS 2G/TJP	3 (50,0)	THEMIS1G/THEMIS2G/PJE1G, EPROC1G/EPROC2G/TJP/PJE2G	7 (41.2)	10 (43.5)	7 (38.9)
Santa Catarina (SC)	e-SAJPG/e-SAJSG	2 (33,3)	e-SAJPG/e-SAJSG/EPROC1G EPROC2G/SEEU	5 (29.4)	7 (30.4)	5 (27.8)
Total (Brazil)		44		97	141	123

e-SAJPG: First Degree Judicial Automation System; e-SAJSG: Second Degree Judicial Automation System; SEEU: Unified Electronic Execution System; PJE1G: 1st Degree Electronic Judicial Process; PJ2G: 2nd Degree Electronic Judicial Process; Projudi: Digital Judicial Process; LIBRA: Judicial Process Management System of the Judicial Branch of Pará; SISCOM: Computerization System of the District Services; SICAP: Process Control and Monitoring System; EPROC1G: First Degree Electronic Process; EPROC2G : Second Degree Electronic Process; SPROC: Procedural System; SAIPRO: Integrated Process Monitoring System Judicial; THEMIS1G: Electronic Petition and Process System 1st degree; THEMIS2G: Electronic Petition and Process System 2nd degree; VEP: System of Virtual Criminal Execution Courts; E-JUS: Electronic Process System; JUDWIN: Unified Procedural Consultation; THEMISWEBRECURSAL: Electronic Petition and Process System 2nd degree; Projudirecursal: Digital Judicial Process 2nd degree; SCPV: Virtual Procedural Control System; QVT: Applications for access to non-graphical systems; SISTJWEB: Graphic Integrated protocol module; E-JUD: Portal of the Judiciary of the State of Espírito Santo; SIEP: System of Criminal Execution; SIAP: Procedural Monitoring System; PJERJ: Electronic Judicial Process of Rio de Janeiro; VEC: Advanced research on physical processes; TJPR: Paraná Court of Justice.

**Table 2:** Description and frequency of systems of the State Courts of Justice, 2021.

Systems in State Courts of Justice^a^	n (%)
Electronic Judicial Process 1st Degree (PJ1G)	17 (13.8)
Electronic Judicial Process 2nd Degree (PJ2G)	13 (10.5)
Digital Judicial Process (Projudi)	16 (13.0)
Unified Electronic Execution System (SEEU)	12 (9.7)
First Degree Judicial Automation System (e-SAJ PG)	10 (8.1)
Electronic Transmission System of First Degree Procedural Acts (E-PROC1G)	3 (2.4)
1st degree Electronic Petition and Process System (THEMIS1G)	3 (2.4)
Electronic First Degree Process (EPROC1G)	3 (2.4)
Electronic Second Degree Process (EPROC2G)	3 (2.4)
Virtual Criminal Enforcement Courts System (VEP)	2 (1.6)
Procedural System (SPROC)	2 (1.6)
2nd degree Electronic Petition and Process System (THEMIS2G)	2 (1.6)
Computerization System for Municipal Services (Siscom)	2 (1.6)
Portal of the Judiciary of the State of Espírito Santo (E-JUD)	1 (0.8)
Electronic Process System (E-JUS)	1 (0.8)
Electronic Administrative Protocol System (E-MAIL)	1 (0.8)
1st degree Electronic Petition and Process System (THEMISWEB)	1 (0.8)
Electronic Judicial Process (Tucujuris)	1 (0.8)
1st Degree Process Monitoring System (SISTJ Gráfico)	1 (0.8)
Judicial Process Management System of the Judiciary of Pará (Libra)	1 (0.8)
Physical proccess consultation portal 1st and 2nd degree	1 (0.8)
2nd degree Electronic Petition and Process System (Themiswebrecursal)	1 (0.8)
Electronic Judicial Process Rio de Janeiro (PJe-RJ)	1 (0.8)
Portal Court of Justice of Paraná (TJPR) State Courts with Physical Processes (on paper)	1 (0.8)
2nd degree Digital Judicial Process (ProjudiRecursal)	1 (0.8)
Applications for access to non-graphical systems (QVT)	1 (0.8)
Integrated Monitoring System of Judicial Processes (SAIPRO)	1 (0.8)
Unified Beta Query	1 (0.8)
Procedural monitoring system Court of Justice Piauí – 2nd instance (e-TJPI)	1 (0.8)
Unified procedural consultation (JUDWIN)	1 (0.8)
Integrated protocol module (SISTJWEB)	1 (0.8)
Procedural Monitoring System (SIAP)	1 (0.8)
Process Control and Monitoring System (SICAP)	1 (0.8)
Electronic procedural search second degree (Second Instance System)	1 (0.8)
Criminal Enforcement System (SIEP)	1 (0.8)
Electronic Judicial Process (Tucujuris Web)	1 (0.8)
Virtual procedural procedure in the Judiciary of Mato Grosso (Sistema Apolo)	1 (0.8)
Virtual Procedural Control System (SCPV)	1 (0.8)
Procedural Control System (Apolo Eletrônico)	1 (0.8)
Advanced Physical Process Search (VEC)	1 (0.8)
First Degree Judicial Automation System (e-SAJ PG5)	1 (0.8)
First Degree Judicial Automation System (e-SAJ SG3)	1 (0.8)
First Degree Judicial Automation System (e-SAJ Digital)	1 (0.8)
First Civil Degree Procedural Consultation (1GCivel)	1 (0.8)
Procedural consultation First Degree Court of criminal executions (1GVEP)	1 (0.8)
Procedural consultation First Criminal Degree (1GCriminal)	1 (0.8)
Second-degree procedural consultation (2G)	1 (0.8)
Court of Justice – Cases (TJP)	1 (0.8)
**Total**	**123 (100)**

^a^ There are 48 types of systems in State Courts of Justice in the country.

The TJ of Acre reported that it uses only electronic processes, presenting three virtual databases, while the TJ of Sergipe indicated having only one database that serves for protocol and monitoring of processes, both physical and virtual. The TJ of Alagoas, Mato Grosso do Sul and Rondônia presented two databases, indicated for both physical and virtual processes, one for first-degree processes and another for second-degree ones. The TJ of Amazonas presented three databases, also indicated for protocol and monitoring of physical and virtual processes.

On the other hand, Rio Grande do Sul, Maranhão and the Distrito Federal (DF) reported bases in each typology, seven in total. The state of Piauí counted two databases for physical processes and six for virtual ones, totaling eight databases. The other TJ have different operating characteristics, from which one can follow the physical and virtual processes (Table 1).

The advancement of electronic systems for the modality of virtual processes to the detriment of the records of physical processes was verified, the fact justifies the repetition of some bases in relation to physical and virtual processes. For example, in the state of Amazonas, there is the E-SAJ and Projudi system, which can both be used to monitor physical and virtual processes.

In the country, 141 databases were identified in the states and DF, 44 (31.2%) for physical processes and 97 (68.8%) for virtual ones (Table 1). There is a predominance of virtual bases in all regions of the country, with emphasis on the Northeast and South regions (Table 1).

A total of 123 databases were found in the TJ, with emphasis on the South and Northeast regions (Table 1). When disregarding the existence of duplication of databases, a total of 48 systems were found throughout the country (Table 2). The most frequent systems were: PJE1G (n = 17; 13.8%), PJE2G (n = 13; 10.5%), Projudi (n = 16; 13%), SEEU (n = 12; 9.7%) and E-SAJ PG (n = 10; 8.1%).

### Stage 2

Responses were obtained to the requests of the TJ of Amapá (AP), Pará (PA), Rondônia (RO), Roraima (RR), Alagoas (AL), Ceará (CE), Maranhão (MA), Rio Grande do Norte (RN), Sergipe (SE), Distrito Federal (DF), Espírito Santo (ES), Minas Gerais (MG), São Paulo (SP) and Paraná (PR).

With the information of 14 participating courts of justice, it was noticed that the branch for protocols of actions related to health is established by the CNJ, based on the System of Unified Procedural Tables (*Sistemas de Tabelas Processuais Unificadas* - TPU), established by Resolution No. 46 of 2007 of the CNJ.

Based on the answer to question [A] according to the classification established by the CNJ table, there are differences in the form of registration and protocol when it comes to public or private health. In addition, the competence to process causes related to public health refers to special courts and courts of the public treasury, which judge civil lawsuits of interest to the state and municipalities, while for private health there are special civil courts. Such details are important for the extraction of reports and future research designs.

In another perspective already brought by question [B], in a common way, the legal operators responsible for the distribution of lawsuits incur error by classifying them in various subjects, such as, for example, “obligation to do^”^ (lawsuit that aims at a provision of one person in relation to another). Although the judicial unit has the possibility to readjust the action, this aspect reflects a competence gap to be filled.

It was also mentioned that there are no specific rules linking competence to matters. The update/correction, however, can be done at any time by internal users of the system, with different profiles, such as protocol, distribution, notaries and/or offices.

Finally, question [C] revealed that in the TJ both the distribution sector and court notaries and the technical support centers of the Judiciary (NAT-Jus) can make adequacy/compatibility, correcting any registration errors.

The TJ of Roraima complemented the information that NAT-Jus “collects” the data, consolidates them, and monitors health actions, especially in tributary and childhood courts, considering the context on request basis. Even if the subject is not “health”, the court analyzes according to the subject, indicating greater rigor in relation to the information available from health actions.

One also found the websites of the TJ do not have a standardized data interface for the provision of processes related to public health, and there are also different obstacles to accessing this type of information. No information was found on how the content of the processes should be made available in the database, leaving at the discretion of each court how to make its data available on the various subjects, such as the processes of judicialization of public health.

Also as a result of this research, it was recognized that the greatest difficulty found in the activity of collecting data on health processes arises from problems in the access models (Judiciary Systems), in the availability and organization of the websites of the various state TJs, i.e., there is no uniformity for proper access, which requires the development of a system that reaches the demands in order to guarantee, in fact, the access of all legal operators, as well as society, including health researchers.

## DISCUSSION

The study allowed additional evidence that the large number of databases that store individual and collective actions involving SUS makes it difficult to carry out more substantiated and comparative analyses. There is a technical limitation for the systematic extraction of data compiled in each system, in addition to the lack of integration between them, compromising the planning, decision-making and development of health research. One also observed that these systems, in more than a hundred, are still of limited access to law operators and researchers, both in the field of law and in the health sciences, particularly Public Health, with potentially negative impacts for academic research in these fields of knowledge.

Although the computerization law of the judicial process No. 11,419 of 2006 and Bill No. 5,828 of 2001 have in their original proposal the prediction that each body of the legal system would develop software necessary for the use of the digital process, creating its own access base and which could be accessed from anywhere on the planet^12^; this administrative autonomy of each TJ, previously without a guiding legislation, generated multiplicity of systems in the courts with lack of uniformity in the databases and interfaces between information in the courts, evidenced in this study.

In 2005, the CNJ, the administrative and procedural control body of the legal system, established the statistics system of the Judiciary Power (Resolution No. of August 4, 2005), making it mandatory for the country’s TJ to send consolidated data on processes and sentences rendered to be centralized in the Council^18^. However, studies show there are limited concrete advances until the completion of this research^19,20^, the different databases limit the production of information that translates reality, reducing the potential for jurisdictional provision^9^. Therefore, compromising the description and broad and precise understanding of the phenomenon of health judicialization, as well as the planning of public policies by the Executive Branch^1,13^.

The automation of the legal system was rethought with the emergence of the electronic judicial process, proposal for integration of databases, and strategic management of information from the judiciary power^21^. It currently represents an important tool standardized by the ordinary Judiciary, a fact verified in this study, based on the finding that it is used by almost 20% of the country’s TJ, reinforcing its strategic implementation as a public policy of the Legal System provided for in CNJ Resolution No. 185 of 2013^22^.

This scenario points out that the use of judicial databases is a powerful tool for faster and more qualified procedural processing^20^ and conducting empirical research in health^1^. However, it was noted that its existence by itself does not guarantee easy access, without being subject to personal, structural and social limitations, in view of the multiplicity, inconstancy and lack of uniformity, in addition to limiting access to the data of the possible analyses, making impossible a series of interfaces between the different bases to overcome possible inconsistencies^1,9,13^.

Another concern refers to the limitation of access for people who do not work in the field of law; based on the findings of this study and experience of linked data collection, it is inferred that the existence of structural barriers consistently limits the wide access to empirical data, objects of research on the judicialization of public health, a fact intensified by the wide variety of databases in the Legal System.

The importance of the proposal to consolidate databases in the Judiciary Branch promotes alignment with the sustainable development objective of Agenda 2030, number 16, which deals with a society with universal access to Justice, with effective and inclusive institutions at all levels^23,24^.

Another important aspect is the classification and protocol of actions related to health (“types” of matters that are the subject of litigation), due to the fact that some courts use as standard the subjects of the CNJ’s unified procedural table. However, there are other ways to proceed to branching, as can be seen from the response of the TJ of Minas Gerais.

The protocol of health actions represents relevant information, considering the large number of studies on health judicialization that require empirical data from the courts for research purposes^1,9,11,13,14^. In this study, the question of whether it is possible to file health actions in other matters was answered “yes” by most courts. Although the use of the managerial entitled “health processes” is regulated, many representatives classify demand differently. For example, in the public treasury there are processes classified with the managerial “obligation to do or not to do”, when in fact they are health processes (supply of medicines). The reasons for this imprecise classification may be related to the recent adoption of new policies for health actions, including the adoption of a specific classification (Box), which has shown limitations due to erroneous registrations referring to the ‘subject’ when distributing the process^1,11,13,14^.

**Box:** Answers on the process of protocol and monitoring of health processes in the databases of the Courts of Justice of the States.

Federative Units (UF)	Question A) In the systems (insert the systems according to the previous question), what is the branch (in the subject tree) to classify and protocol an action that deals with health? Is there a difference in the protocol when it comes to public health and private health?	Question B) Is it possible to carry out a protocol of a process in the competence of the public treasury that is of the health area, without the registration being identified that it is of the health, for example (register as “administrative act/annulment” – but be referring to the supply of medicines)?	Question C) Does the distribution sector make adequacy/compatibility in the registration of actions, correcting any petition errors by the professionals who make the registrations?
Amapá (AP)	Regarding the branch, in the subject tree, to classify and file an action that deals with health, the Tucujuris system – Electronic Judicial Process uses the System of Unified Procedural Tables (TPU), established by Resolution No. 46/2007 of the National Council of Justice (CNJ), available at: https://www.cnj.jus.br/sgt/consulta_publica_classes.php	Yes, the lawyer is the one who classifies/registers among the rites available at the time of the Initial Petition, and it is possible that he/she selects or classifies a rite different from the intended action, and the Secretariat may perform the correction.	The distribution is automatic, performed by the lawyer himself, and after distribution the office realizes the performance and the admissibility examination, and the Secretariat may perform the correction.
Pará (PA)	The Court of Justice of Pará (TJPA) uses the electronic system PJE for the protocol of initial petitions. To register “subject” for an initial process, you must first select the “class” of the action. In view of the class, it is possible to classify the subject among those existing in the system, according to the object of the action. To make a difference between “public health” and “private health”, for the first case, the subject should be selected in the tree of “Administrative Law” and “Health Law”; and for the second, it should be in the tree of “Consumer Law”. In both cases, the one that best identifies the action should be verified as the main subject. We use this opportunity to clarify that nothing prevents other subsidiary matters from being selected. We also emphasize that the initial action protocol is the responsibility of the lawyer, who is responsible for class classification and the subject of the action to be filed, observing the jurisdiction of the court, before the final protocol.	It is possible to perform the protocol of an action without performing the correct classification, because everything depends on the class and subject selected by the lawyer. It should also be noted that the subject tree used by this Court is that provided by the CNJ (Unified Procedural Table Management System – CNJ – https://www.cnj.jus.br/sgt/consulta_publica_assuntos.php). This body is better able to provide the clarifications questioned.	The Distribution Sector does not have the competence to change/correct errors that occurred during the protocol of an action. If any adjustment has to be made, it must be carried out by the respective Registry of the Court.
Rondônia (RO)	Regarding the branching of the subject tree used, the subjects belong to the tree: Health Law. The glossary of TPU explains when it comes to private and public health.	There is a possibility of distribution with any matter that is associated with the jurisdiction of the Public Treasury court.	Users with a secretary director profile are enabled to perform subject corrections.
Roraima (RR)	The system currently widely used in the TJRR is the Projudi (there are still PJE and Siscom, however on a very small scale), so the TJRR adopts as a model of the unified tables of the CNJ, among the subjects or better managerial titles we have: “Health Processes”, it is this managerial that should be used at the time of filing the action to classify the newly filed process.	As for the protocol, although the use of the managerial “Health processes” is regulated, many representatives end up classifying the demand in a different way, for example in the public treasury there are processes classified with the managerial “obligation to do or not to do” when in fact they are health processes, including the supply of medicines. I believe that one of the reasons is the recent adoption of new policies for health actions, including the adoption of a specific classification.	With regard to the adequacy/ compatibility of any misconceptions in the definition of managerial in the register, both the distribution sector, the court registry office and NATJUS can change the classification when any misconceptions are found in the definition of managerial. Finally, it is relevant to note that NATJUS “collects” the statistical data and monitors health actions, especially in tributary and childhood courts, considering each request, i.e., NATJUS confers the daily collection that is distributed in the referred units, even though it is a managerial different from that indicated as “health process”, this nucleus records the process in its statistics since an analysis of the initials of each process is made to ensure the accuracy of the statistics.
Alagoas (AL)	The branch is defined by the CNJ through the Management System of Unified Procedural Tables – (TPU), and applies to all courts in the country. Namely, any branch that is below the subject of code 12,480 (Health Law). About the difference in the protocol: Yes, there is a difference in the registration of these actions. When it comes to public health, the branch used is the subject of code 12,481 (public health), while in relation to private health, the branch of code 12,482 (supplementary health) is used. We should clarify that the CNJ identifies these matters by subject (complement) and not by class (action).	Yes, that is possible. The subject matter is not a mandatory content of the process, so possible misunderstandings may occur.	The distribution sector is not properly in charge of correcting and/or identifying matters, but rather of competence. In this case, the health theme (in the context presented here) is defined by the subject and the competence is the non-criminal – defined by the procedural class. Therefore, the judicial unit that receives the case is in charge of identifying the matter and correcting it, if necessary.
Ceará (CE)	One informed according to resolution §§1 of 09/2019 of the ECJ as well as IN 03/2018 that the protocols were specifically and restricted to the areas of the right to public health, since the tree of Administrative Law is pertinent. However, the National Council of Justice (https://www.cnj.jus.br/sgt/consulta_publica_assuntos.php), promoted a recent change in the national table, including the branch of matters related to “Health Law”, repealing some codes of those that were standardized in the specialization carried out by the ECJ in 2018 and including several others related to the judicialization of private health.	NR	I inform that until the advent of automatic distribution in the Public Treasury Courts or the Civil Courts, the distributor user is authorized to correct the class eventually mistakenly elected by the petitioner.
Maranhão (MA)	The Information Technology Advisory made a search in all the filed processes that contain “Health” in the subject. Thus, processes were found in the following branches: Administrative Law and other matters of Public Law, Health Law, Consumer Law and Tax Law. It can be seen from the definition of the subjects mentioned in the previous table that there is a way to separate public and private health.	Computer Advisory reports that there is the concept of competence in judicial proceedings systems. Cross-referencing health issues (mentioned in the table provided) with the competencies of each process.	The Computer Advisory can report that whenever the distribution departments of the forums receive the determination to correct the assessment of the processes, either by changing the class, competence, inclusion or exclusion of subjects, among other data, this is done in the system.
Rio Grande do Norte (RN)	Health Law - 12,480 and its ramifications - There is a difference in the process record regarding public and private health according to codes provided by the TJ.	It is possible, however, to minimize these misconceptions; the Secretariat for Strategic Management has been continuously disseminating information explaining the proper use of the Unified Procedural Tables. In addition to the Internal Affairs action in its Correction reports, in which the inconsistencies of registrations are pointed out.	Registration errors are as far as possible corrected by the judicial units, there is no specific sector for this purpose. The Court has been preparing a compiled by jurisdiction, in which the classes, subjects and movements appropriate for each jurisdiction are identified, in order to avoid the occurrence of these misconceptions.
Sergipe (SE)	The virtual procedural control system used by this Court is parameterized according to the Unified Procedural Tables of the National Council of Justice and, in the latter, it appears as a branch to classify and file an action that addresses the subject of Health Law (12,480), which has the following subdivisions: 1. Donation and transplantation of organs, tissues or parts (12,521). 2. Genetics/stem cells (12,520).	Yes, it is possible given that the lawyer subscribing to the petition must provide the matter when filing the action under the caput of article 170-F of the Judicial Normative Consolidation with wording amended by Provision No. 22/2010 and, therefore, may be mistaken in the choice of matter.	Article 170-F of the Judicial Normative Consolidation, in its paragraph 5, exposes that the distributor can remedy any inaccuracies and include data indispensable to the registration of the process, always using the initial petition as a parameter. On the other hand, the applicant informs that it sends the above questions to this Court of Justice with a view to making some requests, which are answered below: 1) Verification of the consolidated data in terms of its adequacy to the issues presented and the realities of this court. It was not clear what these consolidated data would be and it is also a subjective question, not recoverable in the virtual procedural control system of this Court.
Sergipe (SE)	3. Mental (12,507), which is subdivided into compulsory hospitalization (12,508), involuntary hospitalization (12,509) and voluntary hospitalization (12,510). 4.Public (12,481), which is subdivided into: 4.1) Supply of inputs (12,485), which is subdivided into wheelchair/bath chair/hospital bed (12,498), dressings/bandage (12,497) and diapers (12,499). 4.2) Supply of medicines (12,484), which is subdivided into oncology (12,496), registered with Anvisa (12,492) and without registration with Anvisa (12,493). It is noteworthy that the matter registered with Anvisa (12,492) is subdivided into non-standard (12,495) and standard (12,494). 4.3) hospitalization/ transfer (12,483), which is subdivided into ward/oncology bed (12,505) and intensive therapy unit (ITU)/intensive care unit (ICU) (12,506). 4.4) Unified Health System (SUS) (12,511), which is subdivided into Social Control and Health Councils (12,518), medical agreement with SUS (12,512), SUS financing (12,513), SUS table adjustment (12,514), transfer of SUS funds (12,515), SUS reimbursement (12,516) and SUS outsourcing (12,517). 4.5) Medical-hospital treatment (12,491), which is subdivided into consultation (12,500), dialysis/hemodialysis (12,504) and surgery (12,501). It is noteworthy that the subject of surgery (12,501) is subdivided into elective (12,502) and urgent (12,503). 4.6) Health and Epidemiological Surveillance (12,519). 5) Supplementary (12,482), which is subdivided into Health plans (12,486) and the latter is subdivided into supply of inputs (12,490), supply of medicines (12,487), contractual adjustment (12,488) and medical-hospital treatment (12,489). In addition, it is worth mentioning that there is no difference regarding the protocol of actions on public or private health, both of which must be filed electronically in compliance with Provision No. 22/2010 and the Judicial Normative Consolidation, both of which are of this Court.	Yes, it is possible given that the lawyer subscribing to the petition must provide the matter when filing the action under the caput of article 170-F of the Judicial Normative Consolidation with wording amended by Provision No. 22/2010 and, therefore, may be mistaken in the choice of matter.	2) If they consider it necessary, they make adjustments with rectification of the data, to match the reality of each court. This process should be confirmed (ratified or rectified) in advance with the answer to the following question: Does this Court confirm the appropriateness of the results presented in the light of your particular reality? This is a subjective data, not recoverable in the virtual procedural control system. 3) Update of the answers of the Court of Justice considering the reality until May 2021, with a view to greater contextualization and comparison with the context of January 2019. In this sense, I request the demarcation of the existence or not of eventual changes and, if any, the specification. The system of electronic petitioning and monitoring of electronic processes remains similar to that used in January 2019, based on Provision No. 22/2010 and Judicial Normative Consolidation.
			In view of the foregoing, therefore, this Civil Division contends that the responses be forwarded to the requesting party of this Electronic Information System (SEI), remaining available for the provision of more information.
Distrito Federal (DF)	The system performs the class and subject configuration to establish and fix the initial competency. For health competence, currently the system associates the class Common Civil Procedure (7), associated with the subjects of the tree of the table SGT of the CNJ of Health Law -12,480, always using the subjects children or father, from the 3rd level. The SGT table has tree branches dealing with public health matters (12,481) and supplementary health matters (12,482). Link to access and consult the SGT table of the CNJ: www.cnj.jus.br/sgt	If the lawyer, attorney-in-fact or public defender, when filing an initial with a class and matter incompatible with civil jurisdiction, the case may be referred to Public Treasury jurisdiction. In these cases, the magistrate will analyze the request and when identifying that it is a health issue, determine the reclassification of the process and the redistribution to the specialized health court.	Considering that the Court of Justice of the Federal District and Territories (TJDFT) implemented the PJe in all jurisdictions (Civil, Criminal, Family, Orphans and Successions, Special Civil and Criminal Courts, Domestic Violence Courts, Childhood Court and others), the distribution service was deactivated, being under the responsibility of the judicial units the prior analysis of the classification of the deeds. TJDFT is also developing a system that uses Artificial Intelligence to assist our users with the correct classification of processes. Currently, the robot is implemented in 11 pilot units.
Espírito Santo (ES)	It uses the System of Unified Procedural Tables (TPU), established by Resolution No. 46/2007 of the National Council of Justice – CNJ, available at: https://www.cnj.jus.br/sgt/consulta_publica_classes.php	It is indeed possible. There are no specific rules linking competence to subjects. The update/correction, however, can be done at any time by internal users of the system, with different profiles, such as protocol, distribution, notaries and/or offices.	The system makes it possible to make the adjustment by the profiles described in item (ii), but we cannot say whether they are carried out.
Minas Gerais (MG)	In the PJe, when distributing the case, the lawyer will choose the class and subject that best suits the intended situation. Several subjects will be presented for distribution and, if there is accumulation of supplementary and public health issues, it will be presented for which competence it wishes to distribute, whether Supplementary Health, State Public Health and Municipal Public Health. When making the choice, the process will be registered in the indicated jurisdiction and directed to the 2nd Civil Court or 2nd State Public Treasury Court or 2nd Municipal Treasury Court, respectively, in the case of for example the district of Belo Horizonte. In Projudi, the lawyer/terminated person will choose the competence first and after the class and subject, so the chances of distribution to the incompetent court are minimized. The competencies that deal with health in Projudi are: Special Civil Courts of Consumption and Special Civil Courts of the Public Treasury.	Yes, because the distribution is made by the lawyer, that is, it is up to him to choose the appropriate competence, class and subject, and can be done mistakenly, as for example, in common justice if the Common Procedure class (7) is chosen with different health issues, the system will never present as a possible competence the health and the process will be registered in a different competence from the health. Consequently, it will be very likely to be distributed to a court that is not competent, in accordance with Resolution 829/2016, for judicial districts that have more than one civil and treasury court. On the other hand, if there is the choice of a health subject (supplementary or public), the PJe system will display among the alternatives of competence the health ones according to the indicated.	The rectification of the registration of the shares is made by the secretariat at the time of issuance of the Screening Certificate, pursuant to art. 195 of Provision No. 355/CGJ/2018, since the distributor does not have access to the processes already distributed. In this situation, currently, even if there is a rectification of the matter, the jurisdiction in which the process was distributed will not be changed, due to the current functioning of the PJe System, which does not occur in PROJUDI. Improvement on this point, alteration/rectification of competence in distributed processes has already been requested from the CNJ and awaits development.
São Paulo (SP)	As for classes and matters, the Court of Justice of São Paulo is adhering to the Unified Procedural Tables of the National Council of Justice, which standardize the petitions of initials in the courts of the country, including the matters that constitute the tree of “code 12,480 – Health Law”.	The system allows the linking of the subjects “Administrative act or Annulment” during the electronic petition of an action under the jurisdiction of the Public Treasury, even if the content of the request is related to health. This is because the subjects codes “11,899 – Administrative Act” and “10,382 – Annulment” belong to the tree of the parent code “9,985 – Administrative Law and Other Matters of Public Law”, which is linked to the competence of the “Public Treasury”; the same happens, for example, with the subjects that belong to the trees of the parent codes “1,156 – Consumer Law”, “8,826 - Civil and Labor Procedural Law”, “9,633 – Child and Adolescent Law”, among others.	The distributions of the initials sent via electronic petition occur automatically, without manual interference from the distributors. If necessary and if it is the magistrate’s understanding, the destination notary of that distributed action may make corrections to the data registered at the time of the petition.
Paraná (PR)	There is no specific treatment for processes classified with health issues in the Projudi system.	The Projudi system has the configuration of enabling classes and subjects by competencies. Once the specified subject is configured in the competence (area of sticks), there would be no impediment to protocolization.	The Projudi system allows the registry to change the class, main and secondary subject of the proceedings at any time.

NR: did not respond; TJCE: Court of Justice of Ceará; TJRR: Court of Justice of Roraima; PJE: Electronic Judicial Process; Anvisa: National Health Surveillance Agency; Projudi: Digital Judicial Process; SGT: System of management of unified procedural tables of the National Council of Justice.

NATJUS: Nuclei of Technical Support to the Judiciary.

Such a scenario points to a risk to the actual number of health judicialization processes in the courts already researched^11^, even though the courts respond that it is possible to make the adjustment. The inaccuracies and inclusions of inaccurate data are questioned, a fact found in the answers provided in the Box.

The limitations of the study refer to the degree of accuracy and systematization of information related to the information systems of the legal system that were formally returned by the TJ. Despite these issues, the national scope and the differentiated approach with interface from different perspectives in the field of Law and Collective Health reinforce its relevance, given the unprecedented and strategic character for the country.

## CONCLUSION

The multiplicity of information systems in the Brazilian Judiciary makes their use more complex for analysis with a view to health research, constituting an obstacle to the more effective updating of the Executive’s public policies. Additional efforts are needed not only to standardize, but also to improve the flows and structure of judicial databases, expanding access and transparency, seeking a transdisciplinary look at research in the fields of Law and Collective Health.

The lack of standardization in the organization of data or public access systems to the various TJs (and their statistical data) hinders the empirical research of health judicialization, which is fundamental for the elaboration of public policies. While the electronic database platforms of the Judiciary are not offered in a unified manner, in an equal manner, the virtualization of the processes will not be able to guarantee the expansion of access to Justice information, on the contrary, it may intensify the disparity between public and private access to Justice.

The future must be the digital process and, if it is to be effective, it must be carefully instituted, with analysis of results, failures and improvements, adapting operators and society as a whole. There is a huge potential for analysis and qualification of public policies, not only linked to the health sector. It is noteworthy that this movement demands strategic social policies that promote unrestricted access by society and health researchers, within the limits of current laws, including based on easily accessible data on their websites.

Therefore, the need for strategic improvement is reinforced to standardize the electronic systems used by the Legal System to govern judicial processes and empirical research in health, given that the configuration adopted limits and hinders research and analysis that can even guide the creation of public policies aimed at identifying and controlling the phenomenon of health judicialization.
