# Supplementary material for: Limits and possibilities for the development of public health research in the legal system
Source: Rev Saude Publica. 2022 Aug 16;56:76. doi: 10.11606/s1518-8787.2022056004203 (PMC9388063; doi:10.11606/s1518-8787.2022056004203)
Supplement: Translation

# Translation: Limites e possibilidades para desenvolvimento de pesquisas em saúde pública no judiciário

**Authors:** Nayla Rochele Nogueira de Andrade, Carlos Francisco Oliveira Nunes, Felipe Braga Albuquerque, Carmem E. Leitão Araújo, Anderson Fuentes Ferreira, Adriana da Silva dos Reis, Alberto Novaes Ramos

**Affiliations:** I Brasil Universidade Federal do Ceará. Faculdade de Medicina. Programa de Pós-Graduação em Saúde Pública. Fortaleza, CE, Brasil; II Brasil Universidade Federal de Santa Catarina. Programa de Pós-Graduação em Engenharia e Gestão do Conhecimento. Florianópolis, Santa Catarina, Brasil; III Brasil Universidade Federal do Ceará. Faculdade de Direito. Departamento de Direito Público. Fortaleza, CE, Brasil; IV Brasil Universidade Federal do Ceará. Faculdade de Direito. Programa de Pós-Graduação em Direito. Fortaleza, CE, Brasil; V Brasil Universidade Federal do Ceará. Faculdade de Medicina. Departamento de Saúde Comunitária. Fortaleza, CE, Brasil; VI Brasil Netherlands Hanseniasis Relief Brasil - NHR Brasil. Fortaleza, CE, Brasil

**Keywords:** Decisões Judiciais, Jurisprudência, Recursos para a Pesquisa, Direito Sanitário, Saúde Pública

## Abstract

**OBJETIVO:**

Caracterizar as bases de dados dos tribunais de justiça do Brasil como potencial ferramenta para a pesquisa em Saúde Coletiva em suas interfaces com as ciências jurídicas.

**MÉTODOS:**

Estudo transversal de natureza quantitativa e descritiva com foco em análise de gestão estratégica e sistemas judiciários.

**RESULTADOS:**

Foram identificadas e analisadas bases de dados utilizadas pela Justiça Comum nas Unidades da Federação para sistematizar processos judiciais. Verificou-se um total de 123 bases de dados nos tribunais de justiça por unidade de federação, com destaque para as regiões Sul e Nordeste, em contraste à região Norte que apresenta menor número de sistemas. Esse grande número de sistemas judiciais limita o acesso a operadores do direito, e dificulta levantamento de evidências por pesquisadores em saúde e, consequentemente, com impactos na gestão estratégica do Poder Executivo. Constatou-se limitações desde o design à extração transparente e democrática de dados pelos próprios usuários, bem como restrita integração entre bases.

**CONCLUSÕES:**

Embora avanços tenham sidos empreendidos nos últimos anos pelos tribunais de justiça para unificação dessas bases, a multiplicidade de sistemas de informação utilizados na Justiça Comum estadual complexifica a gestão do conhecimento, limita o desenvolvimento de pesquisas, mesmo quando realizados por advogados ou pesquisadores da área jurídica, gera lentidão na extração de dados para a gestão pública. Reconhece-se a necessidade de esforços adicionais para a padronização, bem como para aprimoramento dessas bases de dados, ampliando acesso, transparência e integração com vistas a um olhar transdisciplinar entre o campo do Direito e da Saúde Coletiva.

## INTRODUÇÃO

Os caminhos para reflexão e pesquisa sobre potenciais conflitos entre sistema político e jurídico na gestão da saúde pública passam, necessariamente, pelo reconhecimento de aspectos que envolvem distribuição e alocação de recursos escassos na sociedade^1^. Particularmente em um país com a dimensão territorial do Brasil, com graves desigualdades sociais que repercutem em padrões epidemiológicos distintos e transicionais, torna-se ainda mais complexo determinar prioridades no sistema de saúde^2^.

O número de ações no Judiciário requerendo bens e serviços em saúde tem crescido de modo significativo no Brasil, sobretudo após 2007, ensejando o fenômeno da judicialização do direito à saúde pública, que neste trabalho é sinônimo de ações judiciais em face de um ente estatal, demandando bens e/ou serviços em saúde^3^.

O maior envolvimento do Poder Judiciário concernente às políticas de saúde, compõe uma discussão maior sobre a “*judicialização da política*”, expressão equivalente a “*politização da justiça*”, reflexo da sua expansão no processo decisório das democracias contemporâneas. Nessa direção, a judicialização do direito à saúde surge como um conflito entre o sistema institucionalizado de ação política e o sistema jurídico^4^.

A judicialização do direito à saúde tem gerado debates cada vez mais frequentes tendo em vista seus múltiplos usos e sentidos^5^, por um lado reforça a dimensão jurídica da cidadania, materializando um direito assegurado na Constituição Federal de 1988, que em seu Artigo 196, afirma ser a saúde um direito de todos e um dever do Estado^6^; por outro, pode reforçar conflitos na governança federativa do Sistema Único de Saúde, limitando a capacidade do poder executivo de planejar, implantar e acompanhar as políticas de saúde por meio de critérios racionais e equitativos^7^. O caráter individual das intervenções e o privilégio de pessoas com maior conhecimento, recursos financeiros ou outras condições de acesso diferenciado à Justiça também são apontados como problemas críticos^8^.

Por certo, um dos efeitos do fenômeno dessa judicialização é a expansão das interfaces entre pesquisas nos campos das ciências da saúde e jurídicas. Verifica-se aumento no número de pesquisas e artigos publicados sobre o tema, com vistas ao dimensionamento e maior compreensão desse fenômeno^3,9^. Por exemplo, há pesquisas com análise de bases de dados do Judiciário no estado do Rio de Janeiro sobre judicialização da saúde^10,11^, no estado do Ceará sobre o fenômeno com reconhecimento dos limites para análise à luz da epidemiologia^3^, e Distrito Federal com a análise da realidade da judicialização em saúde^12^. Em uma abordagem mais ampliada para o país, o Conselho Nacional de Justiça (CNJ) buscou caracterizar o fenômeno e trazer reflexões adicionais sobre o tema^1,13^.

Ao passo que o fenômeno da judicialização cresce, verifica-se a fragilidade dos sistemas de informação do Judiciário na sistematização de dados e de sistemas de acesso público a vários tribunais com potencial de acesso e utilização dos sistemas de informação. Como resultado, pode impossibilitar ou limitar análises fundamentais para implementação de políticas públicas fundamentadas em evidências científicas, em virtude de limitações e inconsistências entre as bases de dados, além de subdimensionamento dos processos de judicialização da saúde^1,9,11,13^.

As críticas e limitações indicam a necessidade de repensar e conhecer melhor as diferentes bases de dados, muitas das quais sem acesso a pesquisadores^3,11^. O presente estudo tem como objetivo caracterizar as bases de dados dos Tribunais de Justiça (TJ) do Brasil como potencial ferramenta para a pesquisa em Saúde Coletiva em suas interfaces com as ciências jurídicas. O reconhecimento da quantidade e qualidade das informações disponíveis é estratégico para análises mais consistentes e propositivas, o que inclui melhor delimitação do fenômeno da judicialização da política pública de saúde no Brasil.

## MÉTODOS

### Desenho do Estudo

Estudo transversal descritivo de abrangência nacional a partir de dados coletados junto aos Tribunais de Justiça Estaduais do país. O processo de coleta de dados foi realizado entre 2019 e 2021 com identificação e caracterização de bases de dados físicas e virtuais utilizadas para sistematização dos processos judiciais e para reconhecimento do processo de protocolo das ações relacionadas à saúde pública.

O estudo foi realizado em duas etapas, ambas fundamentadas em requerimentos formais de dados e informações junto às ouvidorias dos TJ via preenchimento de formulários eletrônicos disponíveis de cada um, processo complementado por meio de consultas sistemáticas a websites oficiais. Todas as manifestações seguiram as regras disponíveis nos endereços eletrônicos e nas diretrizes internas direcionadas por cada tribunal.

A primeira etapa junto às ouvidorias foi baseada em composição de demanda via e-mail, contato telefônico e formulário eletrônico específico disponível nos sites dos TJ, seguindo protocolos internos específicos em cada Unidade da Federação (UF). O acompanhamento da solicitação era realizado com o número de protocolo gerado no ato da solicitação.

Os TJ deveriam responder a três itens específicos:

- Quais os nomes e as datas de implementação dos sistemas utilizados pelos operadores do direito desta UF para registrar e acompanhar os processos judiciais físicos protocolizados?
- Quais os nomes e as datas de implementação dos sistemas utilizados pelos operadores do direito desta UF para registrar e acompanhar os processos judiciais virtuais protocolizados?
- Alguma observação que o TJ entenda pertinente.

Concluída a primeira etapa, optou-se por complementar os dados obtidos a partir de uma segunda etapa, que consistiu em enviar novas solicitações aos TJ para detalhamento adicional do processo de protocolo de ações que versam sobre saúde pública a partir de três questionamentos:

- Nos sistemas de informação disponíveis, sistemas referidos pelo tribunal na primeira etapa, qual a ramificação, na árvore de assuntos, para classificar e protocolar uma ação que trate de saúde? Existe diferença no protocolo quando se trata de saúde pública e saúde privada?
- É possível realizar o protocolo de um processo na competência da fazenda pública que seja da área da saúde, sem que no cadastro seja identificado que seja da saúde, por exemplo: cadastrar como “ato administrativo/anulação” – mas ser referente à oferta de medicamentos?
- O setor de distribuição faz adequação/compatibilização no cadastro de ações, corrigindo eventuais erros de peticionamento por parte dos profissionais que fazem os cadastros?

### Análise de Dados

Para a primeira etapa, em posse das informações coletadas, os dados foram consolidados e organizados em tabelas, com análise descritiva preliminar. Nos poucos casos em que uma mesma base de dados foi informada pelos TJ, como contendo processos físicos e virtuais, computou-se esse dado apenas uma vez, para evitar superestimativas da realidade local.

Para a segunda etapa sobre o processo de protocolo das ações que versam sobre saúde pública, as três perguntas respondidas pelos TJ foram consolidadas e analisadas descritivamente.

### Aspectos Éticos

Os dados deste estudo são secundários e públicos conforme princípio da publicidade do Art. 5º, inc. LX, da Constituição Federal de 1988, Art. 189 do Código de Processo Civil, e Lei 12.527/11^11^, e outros dispositivos.

Ademais, o projeto foi submetido na Plataforma Brasil, ao comitê de ética em pesquisa da Universidade Federal do Ceará-CEP/UFC/PROPESQ, que teve como parecer, declaração dispondo “*o projeto não se aplica à avaliação do comitê de ética em pesquisa, posto se tratar de pesquisa que utiliza informações de acesso livre e por utilizar banco de dados, cujas informações são agregadas, sem possibilidade de identificação individual, de maneira similar ao disposto na Resolução CNS nº 510, de 07 de abril de 2016*”.

## RESULTADOS

### Etapa 1

No estado do Ceará (CE) os dados foram coletados *in loco*, pela facilidade de acesso da equipe de pesquisadores.

Os TJ do Acre (AC), Amapá (AP), Amazonas (AM), Pará (PA), Rondônia (RO), Roraima (RR), Alagoas (AL), Bahia (BA), Maranhão (MA), Rio Grande do Norte (RN), Distrito Federal (DF), Mato Grosso (MT), Espírito Santo (ES), Minas Gerais (MG), São Paulo (SP) e Rio Grande do Sul (RS) retornaram os contatos via e-mail, correspondendo a 59,2% do total.

Para os TJ do Tocantins (TO), Sergipe (SE), Paraíba (PB), Pernambuco (PE), Piauí (PI), Goiás (GO), Mato Grosso do Sul (MS), Rio de Janeiro (RJ), Paraná (PR) e Santa Catarina (SC), foram realizados contatos telefônicos diretos às ouvidorias, assim como para os setores responsáveis pelo serviço de tecnologia da informação dos tribunais. Adicionalmente, foram realizadas buscas sistematizadas em websites. Em todos os casos, foi possível compor perspectivas aos três itens de interesse nessa etapa.

O resultado da primeira etapa do estudo está sintetizado nas Tabelas 1 e 2. A Tabela 1 caracteriza as bases de dados existentes dos sistemas de informação em cada TJ das UF do país, assim como aquelas que se destinam ao registro de processos físicos (bases antigas) e ao registro de processos virtuais (bases modernas).

**Tabela 1:** Número e especificação por unidade de federação e região do Brasil das bases de dados em Tribunais de Justiça Estaduais, 2021.

Unidade da Federação (UF)	Bases de dados	Processos físicos	Bases de dados	Processos virtuais	Total geral	Total sem duplicidade
	Abreviação	n (%)	Abreviação	n (%)	n (%)	n (%)
**Norte**		9 (20,5)		23 (23,7)	32 (33,0)	26 (26,8)
Acre (AC)	Sem dados	0 (0)	e-SAJPG/e-SAJSG/SEEU	3 (13,0)	3 (9,4)	3 (11,5)
Amapá (AP)	Tucujuris	1 (11,1)	Tucujurisweb/SEEU/PJE1G	3 (13,0)	4 (12,5)	3 (11,5)
Amazonas (AM)	e-SAJ PG/e- SAJ SG/Projudi	3 (33,3)	e-SAJ PG/e -SAJ SG/Projudi	3 (13,0)	6 (18,8)	3 (11,5)
Pará (PA)	Libra	1 (11,1)	PJE1G/PJE2G/SEEU/Projudi	4 (17,4)	5 (15,6)	5 (19,2)
Rondônia (RO)	PJE1G, PJE2G	2 (22,2)	PJE1G, PJE2G	2 (8,7)	4 (12,5)	2 (7,7)
Roraima (RR)	Siscom	1 (11,1)	Projudi, SEEU, PJE1G, PJE2G	4 (17,4)	5 (15,6)	5 (19,2)
Tocantins (TO)	Sicap	1 (11,1)	EPROC1G/EPRO2G/Projudi/SPROC	4 (17,4)	5 (15,6)	5 (19,2)
**Nordeste**		15 (34,1)		32 (33,0)	47 (48,5)	44 (45,4)
Alagoas (AL)	e-SAJ PG/SAJ SG	2 (13,3)	e-SAJ PG/e-SAJ SG	2 (6,3)	4 (8,5)	2 (4,5)
Bahia (BA)	Saipro	1 (6,7)	e-SAJ PG/e-SAJ SG/PJE1G/Projudi	4 (12,5)	5 (10,6)	5 (11,4)
Ceará (CE)	Projudi/SPROC	2 (13,3)	e-SAJPG/e-SAJSG, PJE1, PJE2	4 (12,5)	6 (12,8)	6 (13,6)
Maranhão (MA)	THEMIS1G/THEMIS2G	2 (13,3)	Projudi/VEP/PJE1G/PJE2/SEEU	5 (15,6)	7 (14,9)	7 (15,9)
Paraíba (PB)	E-JUS/VEP	2 (13,3)	PJE1G/PJE2G/Consulta Unificada Beta	3 (9,4)	5 (10,6)	5 (11,4)
Pernambuco (PE)	JUDWIN	1 (6,7)	PJE1G, PJE2G, Projudi, SEEU	4 (12,5)	5 (10,6)	5 (11,4)
Piauí (PI)	Themisweb/Themiswebrecursal	2 (13,3)	Projudi/Projudirecursal/SEEU/e-TJPI PJ1G/PJE2G	6 (18,8)	8 (17,0)	8 (18,2)
Rio Grande do Norte (RN)	e-SAJ PG5, e-SAJ SG3	2 (13,3)	e-SAJ PG Digital, PJE1G, PJE2G	3 (9,4)	5 (10,6)	5 (11,4)
Sergipe (SE)	SCPV	1 (6,7)	SCPV	1 (3,1)	2 (4,3)	1 (2,3)
**Centro-Oeste**		7 (15,9)		12 (12,4)	19 (19,6)	16 (16,5)
Distrito Federal (DF)	QVT/SISTJ Gráfico/SISTJWEB	3 (42,9)	Projudi/PJE1/PJE2/SEEU	4 (33,3)	7 (36,8)	7 (43,8)
Goiás (GO)	Portal consulta processo físico 1º e 2º	1 (14,3)	Projudi/SEEU	2 (16,7)	3 (15,8)	3 (18,8)
Mato Grosso do Sul (MS)	e- SAJPG/e-SAJSG	2 (28,6)	e-SAJPG/e-SAJSG	2 (16,7)	4 (21,1)	2 (12,5)
Mato Grosso (MT)	Sistema Apolo	1 (14,3)	Apolo eletronico/PJE1G/PJE2G Projudi	4 (33,3)	5 (26,3)	4 (25,0)
**Sudeste**		7 (15,9)		13 (13,4)	20 (20,6)	19 (19,6)
Espírito Santo (ES)	Sistema de Segunda Instância EJUD/SIEP	3 (42,9)	Projudi/PJE1G/SEEU	3 (23,1)	6 (30,0)	6 (31,6)
Minas Gerais (MG)	SIAP/Siscom	2 (28,6)	PJE1G/PJE2G/Projudi/SEEU	4 (30,8)	6 (30,0)	6 (31,6)
Rio de Janeiro (RJ)	PJERJ	1 (14,3)	E-mail, Projudi, PJE1G, PJERJ	4 (30,8)	5 (25,0)	4 (21,1)
São Paulo (SP)	VEC	1 (14,3)	e-SAJPG/e-SAJSG	2 (15,4)	3 (15,0)	3 (15,8)
**Sul**		6 (13,6)		17 (17,5)	23 (23,7)	18 (18,6)
Paraná (PR)	Portal TJPR Varas Estatizadas com Processos Físicos (em papel)	1 (16,7)	Projudi 1GCÍVEL/1GVEP/1GCRIMINAL/2G	5 (29,4)	6 (26,1)	6 (33,3)
Rio Grande do Sul (RS)	THEMIS1G/THEMIS 2G/TJP	3 (50)	THEMIS1G/THEMIS2G/PJE1G, EPROC1G/EPROC2G/TJP/PJE2G	7 (41,2)	10 (43,5)	7 (38,9)
Santa Catarina (SC)	e-SAJPG/e-SAJSG	2 (33,3)	e-SAJPG/e-SAJSG/EPROC1G EPROC2G/SEEU	5 (29,4)	7 (30,4)	5 (27,8)
**Total (Brasil)**		44	-	97	141	123

e-SAJPG: Sistema de Automação Judicial do Primeiro Grau; e-SAJSG: Sistema de Automação Judicial do Segundo Grau; SEEU: Sistema Eletrônico de Execução Unificado; PJE1G: Processo Judicial Eletrônico 1ºGrau; PJ2G: Processo Judicial Eletrônico 2º Grau; Projudi: Processo Judicial Digital; LIBRA: Sistema de Gestão de Processos Judiciais do Poder Judiciário do Pará; Siscom: Sistema de Informatização dos Serviços das Comarcas; SICAP: Sistema de Controle e Acompanhamento de Processos; EPROC1G: Processo Eletrônico Primeiro Grau; EPROC2G: Processo Eletrônico Segundo Grau; SPROC: Sistema Processual; SAIPRO: Sistema de Acompanhamento Integrado de Processos Judiciais; THEMIS1G: Sistema de Peticionamento e de Processo Eletrônico 1º grau; THEMIS2G: Sistema de Peticionamento e de Processo Eletrônico 2º grau; VEP: Sistema de Varas de Execuções Penais Virtuais; E-JUS: Sistema de Processo Eletrônico; JUDWIN: Consulta Processual Unificada; THEMISWEBRECURSAL: Sistema de Peticionamento e de Processo Eletrônico 2º grau; Projudirecursal: Processo Judicial Digital 2º grau; SCPV: Sistema de Controle Processual Virtual ; QVT: Aplicativos para acesso a sistemas não gráficos; SISTJWEB: Gráfico Módulo de protocolo integrado; E-JUD: Portal do Poder Judiciário do Estado do Espírito Santo; SIEP: Sistema de Execução Penal; SIAP: Sistema de Acompanhamento Processual; PJERJ: Processo Judicial Eletrônico do Rio de Janeiro; VEC: Pesquisa avançada processos físicos; TJPR: Tribunal de Justiça do Paraná.

**Tabela 2:** Descrição e frequência de sistemas dos Tribunais de Justiça Estaduais, 2021.

Sistemas em Tribunais de Justiça Estaduais^a^	n (%)
Processo Judicial Eletrônico 1ºGrau (PJ1G)	17 (13,8)
Processo Judicial Eletrônico 2º Grau (PJ2G)	13 (10,5)
Processo Judicial Digital (Projudi)	16 (13,0)
Sistema Eletrônico de Execução Unificado (SEEU)	12 (9,7)
Sistema de Automação Judicial do Primeiro Grau (e- SAJ PG)	10 (8,1)
Sistema de Transmissão Eletrônica de Atos Processuais Primeiro Grau (E-PROC1G)	3 (2,4)
Sistema de Peticionamento e de Processo Eletrônico 1º grau (THEMIS1G)	3 (2,4)
Processo Eletrônico Primeiro Grau (EPROC1G)	3 (2,4)
Processo Eletrônico Segundo Grau (EPROC2G)	3 (2,4)
Sistema de Varas de Execuções Penais Virtuais (VEP)	2 (1,6)
Sistema Processual (SPROC)	2 (1,6)
Sistema de Peticionamento e de Processo Eletrônico 2º grau (THEMIS2G)	2 (1,6)
Sistema de Informatização dos Serviços das Comarcas (Siscom)	2 (1,6)
Portal do Poder Judiciário do Estado do Espírito Santo (E-JUD)	1 (0,8)
Sistema de Processo Eletrônico (E-JUS)	1 (0,8)
Sistema de Protocolo Administrativo Eletrônico (E-MAIL)	1 (0,8)
Sistema de Peticionamento e de Processo Eletrônico 1º grau (THEMISWEB)	1 (0,8)
Processo Judicial Eletrônico (Tucujuris)	1 (0,8)
Sistema de Acompanhamento de Processos 1º Grau (SISTJ Gráfico)	1 (0,8)
Sistema de Gestão de Processos Judiciais do Poder Judiciário do Pará (Libra)	1 (0,8)
Portal consulta processo físico 1º e 2º	1 (0,8)
Sistema de Peticionamento e de Processo Eletrônico 2º grau (Themiswebrecursal)	1 (0,8)
Processo Judicial Eletrônico Rio de Janeiro (PJe-RJ)	1 (0,8)
Portal Tribunal Justiça do Paraná (TJPR) Varas Estatizadas com Processos Físicos (em papel)	1 (0,8)
Processo Judicial Digital 2º grau (ProjudiRecursal)	1 (0,8)
Aplicativos para acesso a sistemas não gráficos (QVT)	1 (0,8)
Sistema de Acompanhamento Integrado de Processos Judiciais (SAIPRO)	1 (0,8)
Consulta Unificada Beta	1 (0,8)
Sistema de acompanhamento processual Tribunal de Justiça Piauí – 2ª instância (e-TJPI)	1 (0,8)
Consulta processual unificada (JUDWIN)	1 (0,8)
Módulo de protocolo integrado (SISTJWEB)	1 (0,8)
Sistema de Acompanhamento Processual (SIAP)	1 (0,8)
Sistema de Controle e Acompanhamento de Processos (SICAP)	1 (0,8)
Busca processual eletrônica segundo grau (Sistema de Segunda Instância)	1 (0,8)
Sistema de Execução Penal (SIEP)	1 (0,8)
Processo Judicial Eletrônico (Tucujuris Web)	1 (0,8)
Trâmite processual de forma virtual no Judiciário mato-grossense (Sistema Apolo)	1 (0,8)
Sistema de Controle Processual Virtual (SCPV)	1 (0,8)
Sistema de Controle Processual (Apolo Eletronico)	1 (0,8)
Pesquisa avançada processos físicos (VEC)	1 (0,8)
Sistema de Automação Judicial do Primeiro Grau (e-SAJ PG5)	1 (0,8)
Sistema de Automação Judicial do Primeiro Grau (e-SAJ SG3)	1 (0,8)
Sistema de Automação Judicial do Primeiro Grau (e-SAJ Digital)	1 (0,8)
Consulta processual Primeiro Grau Cível (1GCivel)	1 (0,8)
Consulta processual Primeiro Grau Vara de execuções penais (1GVEP)	1 (0,8)
Consulta processual Primeiro Grau Criminal (1GCriminal)	1 (0,8)
Consulta processual segundo grau (2G)	1 (0,8)
Tribunal de Justiça – Processos (TJP)	1 (0,8)
**Total**	**123 (100)**

^a^ Existem 48 tipos de sistemas em Tribunais de Justiça Estaduais no país.

O TJ do Acre informou que utiliza somente processos eletrônicos, apresentando três bases de dados virtuais, enquanto o TJ de Sergipe indicou ter somente uma base de dados que serve para protocolo e acompanhamento de processos, tanto físicos quanto virtuais. Os TJ de Alagoas, Mato Grosso do Sul e Rondônia apresentaram duas bases de dados, indicadas tanto para processos físicos quanto para processos virtuais, uma para processos de primeiro grau e outra para aqueles de segundo grau. O TJ do Amazonas apresentou três bases de dados, também indicadas para protocolo e acompanhamento de processos físicos e virtuais.

Por outro lado, Rio Grande do Sul, Maranhão e Distrito Federal informaram bases em cada tipologia, sete no total. O estado do Piauí contabilizou duas bases de dados para processos físicos e seis para virtuais, totalizando oito bases. Os demais TJ apresentam diferentes características de funcionamento, a partir das quais se pode acompanhar os processos físicos e virtuais (Tabela 1).

Constatou-se o avanço dos sistemas eletrônicos para a modalidade de processos virtuais em detrimento aos autos de processos físicos, o fato justifica a repetição de algumas bases em relação a processos físicos e virtuais. Por exemplo, no estado do Amazonas, tem-se o sistema E-SAJ e Projudi, que tanto podem ser utilizados para acompanhamento de processos físicos quanto para processos virtuais.

Foram identificadas no país 141 bases de dados nos estados e DF, 44 (31,2%) para processos físicos e 97 (68,8%) para virtuais (Tabela 1). Há um predomínio de bases virtuais em todas as regiões do país, com destaque para as regiões Nordeste e Sul (Tabela 1).

Verificou-se um total de 123 bases de dados nos TJ, com destaque para as regiões Sul e Nordeste (Tabela 1). Ao se desconsiderar a existência de duplicidade de bases de dados, constatou-se um total de 48 sistemas pelo país (Tabela 2). Os sistemas mais frequentes foram: PJE1G (n = 17; 13,8%), PJE2G (n = 13; 10,5%), Projudi (n = 16; 13%), SEEU (n = 12; 9,7%) e E-SAJ PG (n = 10; 8,1%).

### Etapa 2

Obteve-se respostas às solicitações dos TJ do Amapá (AP), Pará (PA), Rondônia (RO), Roraima (RR), Alagoas (AL), Ceará (CE), Maranhão (MA), Rio Grande do Norte (RN), Sergipe (SE), Distrito Federal (DF), Espírito Santo (ES), Minas Gerais (MG), São Paulo (SP) e Paraná (PR) (Quadro).

**Quadro:** Respostas sobre o processo de protocolo e acompanhamento de processos de saúde nas bases de dados dos Tribunais de Justiça dos Estados.

Unidade da Federação (UF)	Pergunta A) Nos sistemas (inserir os sistemas conforme pergunta anterior), qual a ramificação (na árvore de assuntos) para classificar e protocolizar uma ação que trate de saúde? Existe diferença no protocolo quando se trata de saúde pública e saúde privada?	Pergunta B) É possível realizar um protocolo de um processo na competência da fazenda pública que seja da área da saúde, sem que no cadastro seja identificado que o mesmo é da saúde, por exemplo (cadastrar como “ato administrativo/anulação” – mas ser referente a oferta de medicamentos)?	Pergunta C) O setor de distribuição faz adequação/compatibilização no cadastro de ações corrigindo eventuais erros de peticionamento dos profissionais que fazem os cadastros?
Amapá (AP)	Referente à ramificação, na árvore de assuntos, para classificar e protocolar uma ação que trate de saúde, o sistema Tucujuris – Processo Judicial Eletrônico utiliza o Sistema de Tabelas Processuais Unificadas (TPU), instituída pela Resolução no 46/2007 do Conselho Nacional de Justiça (CNJ), disponível em: https://www.cnj.jus.br/sgt/consulta_publica_classes.php	Sim, o advogado é quem classifica/cadastra entre os ritos disponíveis no momento da Petição Inicial, sendo possível que o mesmo selecione ou classifique um rito diferente da ação pretendida, podendo, a Secretaria realizar a correção.	A distribuição é automática realizada pelo próprio advogado, após a distribuição o gabinete realiza a atuação e o exame de admissibilidade, podendo, a Secretaria realizar a correção.
Pará (PA)	O Tribunal de Justiça do Pará (TJPA) utiliza o sistema eletrônico PJE para o protocolo de petições iniciais. Para que se possa cadastrar “assunto” para um processo inicial, primeiramente se terá que selecionar a “classe” da ação. Diante da classe, é que poderá se classificar o assunto, dentre os existentes no sistema, conforme o objeto da ação. Para se fazer diferença entre “saúde pública” e “saúde privada”, deverá ser selecionado, para o primeiro caso, assunto na árvore de “Direito Administrativo” e “Direito da Saúde”; e, para o segundo, deverá ser na árvore de “Direito do Consumidor”. Em ambos os casos, deverá ser verificado aquele que identifique melhor a ação, como assunto principal. Esclarecemos, na oportunidade, que nada impede de serem selecionados outros assuntos subsidiários. Ressaltamos, ainda, que o protocolo de ações iniciais está a cargo do advogado, que é responsável pela classificação da classe e do assunto da ação a ser ajuizada, observando a competência da vara, antes do protocolo final.	É possível realizar o protocolo de uma ação sem que seja realizada a classificação correta, pois tudo depende da classe e do assunto selecionados pelo advogado. Frisa-se, também, que a árvore de assuntos, utilizada por este Tribunal, é a disponibilizada pelo CNJ (Sistema de Gestão de Tabelas Processuais Unificadas – CNJ – https://www.cnj.jus.br/sgt/consulta_publica_assuntos.php). Órgão aquele que está melhor capacitado a prestar os esclarecimentos questionados.	O Setor de Distribuição não possui competência para alterar/corrigir erros ocorridos por ocasião do protocolo de uma ação. Caso tenha que ser realizada alguma adequação, esta deverá ser procedida pela Secretaria da Vara respectiva.
Rondônia (RO)	Sobre a ramificação da árvore de assuntos utilizada, são os assuntos da árvore: Direito da Saúde. No glossário da TPU explica quando se trata de saúde privada e pública.	Há possibilidade de distribuição com qualquer assunto que esteja associado à competência do juizado da Fazenda Pública.	Os usuários com perfil de diretor de secretaria estão habilitados para realizar correções de assuntos.
Roraima (RR)	O sistema amplamente empregado atualmente no TJRR é o Projudi (ainda existem PJE e Siscom, contudo numa escala muito reduzida), sendo assim o TJRR adota como modele de as tabelas unificadas do CNJ, dentre os assuntos ou melhor títulos de gerenciais temos: “Processos de Saúde”, é essa gerencial que dever ser utilizada no momento da propositura da ação para classificar o processo recém protocolado.	Quanto ao protocolo, em que pese estar regulamentado o uso da gerencial “Processos de saúde” muitos representantes acabam classificando a demanda de forma diversa, por exemplo na fazenda pública existem processos classificados com a gerencial “obrigação de fazer ou não fazer” quando na verdade tratam-se de processos de saúde, inclusive de fornecimento de medicamentos. Acredito que um dos motivos seja a recente adoção de novas políticas para ações de saúde, inclusive a adoção de classificação específica.	No que se refere à adequação/ compatibilização de eventuais equívocos na definição da gerencial no cadastro, tanto o setor de distribuição, quanto o cartório da vara e o NATJUS podem fazer a alteração da classificação quando constatados eventuais equívocos na definição da gerencial. Por fim, é relevante consignar que o NATJUS “coleta” os dados estatísticos e faz o acompanhando das ações de saúde, especialmente nas varas e juizados da fazenda e da infância, considerando pedido a pedido, ou seja, o NATJUS confere o acervo diário que é distribuído nas referidas unidades, mesmo constando gerencial diversa daquele indicada como “processo de saúde” este núcleo registra o processo em suas estatísticas visto que é feita uma análise das iniciais de cada processo para garantir a precisão das estatísticas.
Alagoas (AL)	A ramificação é mesmo definida pelo CNJ por meio do Sistema de Gestão das Tabelas Processuais Unificadas – (TPU), e se aplica a todos os tribunais do país. A saber, toda a ramificação que estiver abaixo do assunto de código 12.480 (Direito da Saúde). Da diferença no protocolo: Sim, existe diferença no cadastramento dessas ações. Quando se trata de saúde pública a ramificação utilizada é a do assunto de código 12.481 (saúde pública), já em relação à saúde privada se utiliza a ramificação do código 12.482 (saúde suplementar). Esclarecendo que o CNJ identifica essas matérias pelo assunto (complemento) e não pela classe (ação).	Sim, é possível. O assunto não é um conteúdo obrigatório do processo, por isso pode acontecer eventuais equívocos.	O setor de distribuição não se encarrega propriamente de fazer a correção e/ou identificação dos assuntos e sim da competência. No caso, o tema saúde (no contexto aqui apresentado) é definido pelo assunto e a competência é a não criminal – definida pela classe processual. Sendo assim, a unidade judiciária que recebe o processo se encarrega de identificar o assunto e corrigi-lo, caso necessário.
Ceará (CE)	Informou conforme a resolução §§1º da 09/2019 do TJCE bem como a IN 03/2018 que os protocolos se davam especifica e restritamente às áreas do direito à saúde pública, posto que pertinentes a árvore do Direito Administrativo. Contudo, O Conselho Nacional de Justiça (https://www.cnj.jus.br/sgt/consulta_publica_assuntos.php), promoveu alteração recente na tabela nacional, incluindo o ramo de assuntos atinentes ao “Direito da Saúde”, revogando alguns códigos daqueles que foram normatizados na especialização havida pelo TJCE em 2018 e incluindo vários outros relativos à judicialização da saúde privada.	NR	Informo que até o advento da distribuição automática nas Varas de Fazenda Pública ou das Varas Cíveis, o usuário distribuidor tem autorização para corrigir a classe eventualmente eleita com equívoco pelo peticionante.
Maranhão (MA)	A Assessoria de Informática fez uma busca em todos os processos protocolados que contenham “Saúde” no assunto. Desta forma, encontrou-se processos nas seguintes ramificações: Direito Administrativo e outras matérias de Direito Público, Direito da Saúde, Direito do Consumidor e Direito Tributário. Verifica-se pela definição dos assuntos mencionados na tabela anterior que há como separar saúde pública e saúde privada.	Assessoria de Informática relata que há o conceito de competência nos sistemas de processos judiciais. Fazendo um cruzamento dos assuntos relativos à saúde (mencionados na tabela fornecida) com as competências de cada processo.	A Assessoria de Informática pode relatar que sempre que as secretarias de distribuição dos fóruns recebem a determinação para correção de retificar a autuação dos processos, seja pela mudança de classe, competência, inclusão ou exclusão de assuntos, dentre outros dados, isto é feito no sistema.
Rio Grande do Norte (RN)	Direito da Saúde - 12.480 e suas ramificações - Existe diferença no cadastro de processo quanto a saúde pública e privada conforme códigos disponibilizados pelo TJ.	É possível, todavia para minimizar esses equívocos a Secretaria de Gestão Estratégica, vem divulgando continuamente informativos explicando o uso adequado das Tabelas Processuais Unificadas. Além da ação da Corregedoria em seus relatórios de Correição, no qual são apontadas as inconsistências de cadastros.	Os erros de cadastro são na medida do possível corrigidos pelas unidades judiciárias, não há um setor específico para esse fim. O Tribunal vem elaborando um compilado por competência, no qual são identificadas as classes, assuntos e movimentos adequados para cada competência, visando evitar a ocorrência desses equívocos.
Sergipe (SE)	O sistema de controle processual virtual utilizado por este Tribunal está parametrizado de acordo comas Tabelas Processuais Unificadas do Conselho Nacional de Justiça e nestas últimas consta como ramificação para classificar e protocolar uma ação que trate de saúde o assunto Direito da Saúde (12.480), o qual possui as seguintes subdivisões: 1. Doação e transplante de órgãos, tecidos ou partes (12.521). 2. Genética/células tronco (12.520).	Sim, é possível tendo em vista que o advogado subscritor da petição deve fornecer o assunto ao ajuizar a ação nos termos do caput do artigo 170-F da Consolidação Normativa Judicial com redação alterada pelo Provimento n° 22/2010 e, portanto, pode equivocar-se na escolha do assunto.	O artigo 170-F da Consolidação Normativa Judicial, em seu parágrafo 5°, expõe que o distribuidor pode sanar eventuais incorreções e incluir dados indispensáveis ao cadastro do processo, sempre usando como parâmetro a petição inicial. Por outro lado, a requerente informa que envia as perguntas acima a este Tribunal de Justiça com vistas a realizar algumas solicitações, as quais são respondidas abaixo: 1) Verificação dos dados consolidados em termos de sua adequação frente às questões apresentadas e as realidades deste tribunal. Não ficou claro quais seriam esses dados consolidados e trata-se também de um questionamento de caráter subjetivo, não recuperável no sistema de controle processual virtual deste Tribunal.
Sergipe (SE)	3. Mental (12.507), sendo este subdividido nos assuntos internação compulsória (12.508), internação involuntária (12.509) e internação voluntária (12.510). 4.Pública (12.481), sendo este subdividido em: 4.1) Fornecimento de insumos (12.485), o qual é subdividido em cadeira de rodas/cadeira de banho/cama hospitalar (12.498), curativos/bandagem (12.497) e fraldas (12.499). 4.2) Fornecimento de medicamentos (12.484), o qual é subdividido em oncológico (12.496), registrado na Anvisa (12.492) e sem registro na Anvisa (12.493). Vale ressaltar que o assunto registrado na Anvisa (12.492) é subdividido em não padronizado (12.495) e padronizado (12.494). 4.3) internação/ transferência hospitalar (12.483), o qual é subdividido em leito de enfermaria/ leito oncológico (12.505) e unidade de terapia intensiva (UTI)/unidade de cuidados intensivos (UCI) (12.506). 4.4) Sistema Único de Saúde (SUS) (12.511), o qual é subdividido em Controle Social e Conselhos de Saúde (12.518), convênio médico com o SUS (12.512), financiamento do SUS (12.513), reajuste da tabela do SUS (12.514), repasse de verbas do SUS (12.515), ressarcimento do SUS (12.516) e terceirização do SUS (12.517). 4.5) Tratamento médico-hospitalar (12.491), o qual é subdividido em consulta (12.500), diálise/hemodiálise (12.504) e cirurgia (12.501). Vale ressaltar que o assunto cirurgia (12.501) é subdividido em eletiva (12.502) e urgência (12.503). 4.6) Vigilância Sanitária e Epidemiológica (12.519). 5) Suplementar (12.482), o qual se subdivide em planos de Saúde (12.486) e este último se subdivide em fornecimento de insumos (12.490), fornecimento de medicamentos (12.487), reajuste contratual (12.488) e tratamento médico-hospitalar (12.489). Ademais, vale ressaltar que não existe diferença quanto ao protocolo de ações acerca de saúde pública ou privada, devendo ambas serem protocoladas de forma eletrônica em observância ao Provimento n° 22/2010 e à Consolidação Normativa Judicial, ambos deste Tribunal.	Sim, é possível tendo em vista que o advogado subscritor da petição deve fornecer o assunto ao ajuizar a ação nos termos do caput do artigo 170-F da Consolidação Normativa Judicial com redação alterada pelo Provimento n° 22/2010 e, portanto, pode equivocar-se na escolha do assunto.	2) Caso considerem necessário, a realização de ajustes com retificação dos dados, de modo a condizer com a realidade de cada tribunal. Este processo deverá ser confirmado (ratificação ou retificação) previamente com a resposta à seguinte questão: Este Tribunal confirma a adequação dos resultados apresentados à luz da sua realidade em particular? Trata-se de um dado de caráter subjetivo, não recuperável no sistema de controle processual virtual. 3) Atualização das respostas do Tribunal de Justiça considerando-se a realidade até maio de 2021, com vistas à maior contextualização e comparação com o contexto de janeiro de 2019. Neste sentido, solicito a demarcação da existência ou não de mudanças eventuais e caso existam, a especificação. O sistema de peticionamento eletrônico e acompanhamento de processos eletrônicos permanece semelhante ao utilizado em janeiro de 2019, com fundamento no Provimento n° 22/2010 e na Consolidação Normativa Judicial.
			Diante do exposto, portanto, esta Divisão Cível pugna que as respostas sejam encaminhadas à parte requerente do presente Sistema Eletrônico de Informações (SEI), permanecendo à disposição para o fornecimento de mais informações.
Distrito Federal (DF)	O sistema realiza a configuração de classe e assunto para estabelecer e fixar a competência inicial. Para a competência de saúde, hoje o sistema associa a classe Procedimento Comum Cível (7), associada aos assuntos da árvore da tabela SGT do CNJ de Direito da Saúde -12.480, sempre utilizando os assuntos filhos ou pai, a partir do 3º nível. A tabela SGT tem as ramificações da árvore que tratam de assuntos de saúde pública (12.481) e assuntos de saúde suplementar (12.482). Link para acesso e consulta da tabela SGT do CNJ: www.cnj.jus.br/sgt	Caso o advogado, procurador ou defensor público, ao protocolar uma inicial com uma classe e assunto incompatível com a competência cível, o processo poderá ser encaminhado a competência de Fazenda Pública. Nestes casos, o magistrado analisará o pedido e ao identificar que se trata de tema de saúde, determinará a reclassificação do processo e a redistribuição para a vara especializada de saúde.	Tendo em vista que o Tribunal de Justiça do Distrito Federal e dos Territórios (TJDFT) implementou o PJe em todas as competências (Cível, Criminal, Família, Órfãos e Sucessões, Juizados Especais Cíveis e Criminais, Juizados de Violência Doméstica, Vara da Infância e outras), o serviço de distribuição foi desativado, ficando sob responsabilidades das unidades judiciais a análise prévia da classificação dos feitos. O TJDFT também está desenvolvendo um sistema que utiliza Inteligência Artificial para auxiliar nossos usuários com a correta classificação dos processos. Atualmente, o robô está implementado em 11 unidades piloto.
Espírito Santo (ES)	Utiliza o Sistema de Tabelas Processuais Unificadas (TPU), instituída pela Resolução no 46/2007 do Conselho Nacional de Justiça – CNJ, disponível em: https://www.cnj.jus.br/sgt/consulta_publica_classes.php	É possível. Não existem regras específicas que vinculem competência com assuntos. A atualização/correção, entretanto, pode ser feita a qualquer momento por usuários internos do sistema, com perfis diversos como protocolo, distribuição, cartórios e/ou gabinetes.	O sistema possibilita que seja feita a adequação pelos perfis descritos no item (ii), mas não podemos afirmar se são realizadas.
Minas Gerais (MG)	No PJe ao distribuir o processo o advogado escolherá a classe e assunto que melhor se adéqua à situação pretendida. Serão apresentados diversos assuntos para distribuição e, caso haja cumulação de assuntos de saúde suplementar e pública será apresentada para qual competência ele deseja distribuir, se Saúde Suplementar, Saúde Pública Estadual e Saúde Pública Municipal. Ao efetuar a escolha o processo será cadastrado na competência indicada e direcionado para a 2ª Vara Cível ou 2ª Vara da Fazenda Pública Estadual ou 2ª Vara da Fazenda Municipal, respectivamente, em se tratando por exemplo da comarca de Belo Horizonte. Já no Projudi o advogado/atermado escolherá a competência primeiro e após a classe e assunto, assim as chances da distribuição para a vara incompetente ficam minimizadas. As competências que tratam de saúde no Projudi são: Juizados Especiais Cíveis do Consumo e Juizados Especiais Cíveis da Fazenda Pública.	Sim, pois a distribuição é feita pelo advogado, ou seja, cabe a ele escolher a competência, classe e assunto adequados, podendo ser feito equivocadamente, como por exemplo, na justiça comum se for escolhida a classe Procedimento Comum (7) com assuntos diversos de saúde, o sistema jamais apresentará como competência possível a de saúde e o processo será cadastrado em competência distinta da de saúde. Consequentemente, terá grande probabilidade de ser distribuído para vara que não é competente, de acordo com o que determina a Resolução 829/2016, isso para comarcas que possuem mais de uma vara cível e da fazenda. Em contrapartida, se houver a escolha de um assunto de saúde (suplementar ou pública), o sistema PJe exibirá dentre as alternativas de competência as de saúde de acordo com o indicado.	A retificação do cadastro das ações é feita pela secretaria no momento de expedição da Certidão de Triagem, nos termos do art. 195 do Provimento nº 355/CGJ/2018, pois o distribuidor não tem acesso aos processos já distribuídos. Nessa situação, atualmente, ainda que haja a retificação do assunto a competência em que o processo foi distribuído não será alterada, isso em razão do atual funcionamento do Sistema PJe, o que não ocorre no Projudi. A melhoria quanto a esse ponto, alteração/retificação da competência em processos distribuídos já foi solicitada ao CNJ e aguarda desenvolvimento.
São Paulo (SP)	Quanto às classes e assuntos, o Tribunal de Justiça de São Paulo está aderente às Tabelas Processuais Unificadas do Conselho Nacional de Justiça, que padronizam os peticionamentos de iniciais nos tribunais do país, incluindo os assuntos que constituem a árvore do “código 12.480 – Direito da Saúde”.	O sistema permite a vinculação dos assuntos “Ato administrativo ou Anulação” durante o peticionamento eletrônico de uma ação na competência da Fazenda Pública, mesmo que o conteúdo do pedido esteja relacionado à saúde. Isto ocorre porque os assuntos códigos “11.899 – Ato administrativo” e “10.382 – Anulação” pertencem à árvore do código pai “9.985 – Direito Administrativo e Outras Matérias de Direito Público”, que está vinculado à competência da “Fazenda Pública”; o mesmo acontece, por exemplo com os assuntos que pertencem as árvores dos códigos pai “1.156 – Direito do Consumidor”, “8.826 – Direito Processual Civil e do Trabalho”, “9.633 – Direito da Criança e do Adolescente”, dentre outros.	As distribuições das iniciais encaminhadas via peticionamento eletrônico ocorrem de maneira automática, sem interferência manual dos distribuidores. Caso haja necessidade e se for do entendimento do magistrado, o cartório de destino daquela ação distribuída poderá efetivar as correções dos dados cadastrados quando do peticionamento.
Paraná (PR)	Não há tratamento específico para processos classificados com assuntos do ramo de saúde no sistema Projudi.	O sistema Projudi possui a configuração de habilitação de classes e assuntos por competências. Estando o assunto especificado configurado na competência (área de varas), não haveria impedimento à protocolização.	O sistema Projudi permite ao cartório alterar a classe, assunto principal e secundários dos processos a qualquer momento.

NR: não respondeu; TJCE: Tribunal de Justiça do Ceará; TJRR: Tribunal de Justiça de Roraima; PJE: Processo Judicial Eletrônico; Anvisa: Agência Nacional de Vigilância Sanitária; Projudi: Processo Judicial Digital; SGT: Sistema de gestão de tabelas processuais unificadas do Conselho Nacional de Justiça.

NATJUS: Núcleos de Apoio Técnico ao Judiciário.

Com as informações de 14 tribunais de justiça participantes, percebeu-se que a ramificação para protocolos de ações relacionadas à saúde é estabelecida pelo CNJ, fundamentado no Sistema de Tabelas Processuais Unificadas (TPU), instituída pela Resolução n^o^ 46 de 2007 do CNJ.

Com base na resposta à pergunta [A] segundo classificação estabelecida pela tabela do CNJ, há diferenças na forma de registro e protocolo quando se trata de saúde pública ou privada. Além disso, a competência para processamento de causas relacionadas à saúde pública remete-se a varas e juizados especiais da fazenda pública, que julga processos cíveis de interesse do estado e municípios, enquanto para a saúde privada há varas e juizados especiais cíveis. Tais detalhamentos são importantes para extração de relatórios e futuros delineamentos de pesquisa.

Em outra perspectiva já trazida pela pergunta [B], de forma corriqueira, os operadores do direito responsáveis pela distribuição das ações judiciais incorrem em erro classificando-as em assunto diversos, como, por exemplo, “obrigação de fazer” (ação judicial que objetiva uma prestação de uma pessoa em relação a outra). Embora a unidade judicial tenha possibilidade de readequar a ação, esse aspecto reflete uma lacuna de competência a ser suprida.

Mencionou-se ainda, que não existem regras específicas que vinculem a competência aos assuntos. A atualização/correção, entretanto, pode ser feita a qualquer momento por usuários internos do sistema, com perfis diversos, como protocolo, distribuição, cartórios e/ou gabinetes.

Por fim, a pergunta [C] revelou que nos TJ tanto o setor de distribuição quanto os cartórios da vara e os núcleos de apoio técnico do Poder Judiciário (NAT-Jus) podem fazer adequação/compatibilização, corrigindo eventuais erros cadastrais.

O TJ de Roraima complementou a informação de que o NAT-Jus “coleta” os dados, consolida-os, e faz acompanhamento das ações de saúde, especialmente nas varas e juizados da fazenda e infância, considerando o contexto, pedido a pedido. Mesmo que o assunto não seja “saúde” o tribunal analisa de acordo com o assunto, indicando maior rigor em relação às informações disponibilizadas das ações de saúde.

Constatou-se ainda que os websites dos TJ não dispõem de uma interface de dados padronizada para disponibilização de processos relativos à saúde pública, havendo também obstáculos distintos para o acesso a esse tipo de informação. Não foram encontradas informações sobre como o conteúdo dos processos deve ser disponibilizado na base de dados, ficando a critério de cada tribunal a forma como disponibiliza seus dados sobre os variados assuntos, como os processos da judicialização da saúde pública.

Ainda como resultado desta pesquisa, reconheceu-se que a maior dificuldade encontrada na atividade de coleta de dados sobre processos de saúde decorre de problemas nos modelos de acesso (Sistemas Judiciários), na disponibilização e organização dos websites dos vários TJ estaduais, ou seja, não há uniformidade para o devido acesso, o que requer o desenvolvimento de um sistema que alcance as demandas visando garantir, de fato, o acesso de todos os operadores do direito, bem como da sociedade, incluindo pesquisadores da saúde.

## DISCUSSÃO

O estudo possibilitou evidências adicionais de que o grande número de bases de dados que armazenam ações individuais e coletivas envolvendo o SUS dificulta a realização de análises mais consubstanciadas e comparativas. Há limitação técnica para a extração sistemática de dados compilados em cada sistema, além de não haver integração entre eles, comprometendo o planejamento, a tomada de decisão e o desenvolvimento de pesquisas em saúde. Observou-se ainda que esses sistemas, em número superior à centena, ainda são de acesso limitado aos operadores do direito e pesquisadores, tanto no campo do direito quanto das ciências da saúde, particularmente a Saúde Coletiva, com impactos potenciais negativos para a pesquisa acadêmica nesses campos do conhecimento.

Apesar de a lei de informatização do processo judicial n^o^ 11.419 de 2006 e o projeto de Lei n^o^ 5.828 de 2001 terem em sua proposta original a previsão de que cada órgão do poder judiciário desenvolveria softwares necessários à utilização do processo digital criando sua própria base de acesso e que pudesse ser acessado de qualquer lugar do planeta^12^, essa autonomia administrativa de cada TJ, previamente sem uma legislação orientadora, gerou multiplicidade de sistemas nos tribunais com falta de uniformização nas bases de dados e de interfaces entre as informações nos tribunais, evidenciado neste estudo.

Em 2005, o CNJ, órgão de controle da atuação administrativa e processual do Poder Judiciário, estabeleceu o sistema de estatística do Poder Judiciário (Resolução n^o^ 4 de agosto de 2005), tornando obrigatório que TJ do país enviassem dados consolidados de processos e sentenças prolatadas para serem centralizados no Conselho^18^. Todavia, estudos mostram que há limitados avanços concretos até a finalização desta pesquisa^19,20^, as diferentes bases de dados limitam a produção de informações que traduzam a realidade, reduzindo o potencial de prestação jurisdicional^9^. Portanto, comprometendo a descrição e entendimento amplo e preciso do fenômeno da judicialização da saúde, assim como o planejamento nas políticas públicas por parte do Poder Executivo^1,13^.

A automação do judiciário foi repensada com o surgimento do processo judicial eletrônico, proposta de integração das bases de dados e gerenciamento estratégico das informações do judiciário^21^. Atualmente representa importante ferramenta uniformizada pelo Judiciário comum, fato verificado neste estudo, a partir da constatação de que é utilizado por quase 20% dos TJ do país, reforçando a sua implantação estratégica como política pública do Judiciário prevista na Resolução CNJ n^22^.

Tal cenário aponta que o uso de bases de dados do judiciário é uma ferramenta potente para tramitação processual mais célere e qualificada^20^ e realização de pesquisas empíricas em saúde^1^. Contudo, notou-se que sua existência por si não garante o fácil acesso, sem estar sujeita a limitações pessoais, estruturais e sociais, tendo em vista a multiplicidade, inconstância e falta de uniformização, além da limitação de acesso aos dados das análises possíveis, inviabilizando uma série de interfaces entre as distintas bases para superar eventuais inconsistências^1,9,13^.

Outra preocupação remete à limitação ao acesso de pessoas que não têm atuação no campo do direito; com base nos achados deste estudo e experiência da coleta de dados vinculada, infere-se que a existência das barreiras estruturais limita de modo consistente o amplo acesso aos dados empíricos, objetos de pesquisas sobre a judicialização da saúde pública, fato intensificado pela grande variedade de bases de dados no Judiciário.

A importância da proposta de consolidação das bases de dados no Poder Judiciário promove o alinhamento ao objetivo de desenvolvimento sustentável da Agenda 2030, número 16 que versa sobre uma sociedade com acesso universal à Justiça, com instituições eficazes e inclusivas em todos os níveis^23,24^.

Outro aspecto importante é classificação e protocolo de ações relacionadas à saúde (“tipos” de matérias que são objeto de litígio), pelo fato de alguns tribunais utilizarem como padrão os assuntos da tabela processual unificada do CNJ. Contudo, há outras modalidades de se proceder à ramificação, como se constata da resposta do TJ de Minas Gerais.

O protocolo das ações de saúde representa informação relevante, levando-se em conta o grande número de pesquisas sobre judicialização da saúde que requerem junto aos tribunais dados empíricos para fins de pesquisa^1,9,11,13,14^. Neste estudo, à indagação se é possível protocolar ações de saúde em outros assuntos, obteve-se como resposta “sim” pela maioria dos tribunais. Embora regulamentado o uso da gerencial intitulada “processos de saúde”, muitos representantes classificam a demanda de forma diversa. Por exemplo, na fazenda pública existem processos classificados com a gerencial “obrigação de fazer ou não fazer”, quando na verdade tratam-se de processos de saúde (fornecimento de medicamentos). Os motivos para essa imprecisa classificação podem estar relacionados à recente adoção de novas políticas para ações de saúde, inclusive a adoção de classificação específica (Quadro), que tem demonstrado limitações em virtude de cadastros errôneos referente ao ‘assunto’ quando da distribuição do processo^1,11,13,14^.

Tal cenário aponta um risco para o real número de processos de judicialização da saúde nos tribunais já pesquisados^11^, mesmo os tribunais respondendo que é possível fazer a adequação. Questionam-se as incorreções e inclusões de dados imprecisos, fato constatado nas respostas dispostas no Quadro.

As limitações do estudo remetem-se ao grau de precisão e de sistematização das informações relacionadas aos sistemas de informação do judiciário que foram retornadas formalmente pelos TJ. A despeito dessas questões, a abrangência nacional e a abordagem diferenciada com interface de diferentes perspectivas do campo do Direito e da Saúde Coletiva reforça a sua relevância, dado o caráter inédito e estratégico para o país.

## CONCLUSÃO

A multiplicidade de sistemas de informação no Judiciário brasileiro complexifica a sua utilização para análises com vistas à pesquisa em saúde, consistindo em óbice à atualização mais eficaz das políticas públicas do Executivo. Reconhece-se a necessidade de esforços adicionais não apenas para a padronização, mas também para aprimoramento dos fluxos e da estrutura das bases de dados judiciais, ampliando o acesso e a transparência, buscando um olhar transdisciplinar em pesquisas nos campos do Direito e da Saúde Coletiva.

A ausência de padronização na organização de dados ou de sistemas de acesso públicos aos vários TJ (e seus dados estatísticos) dificulta a pesquisa empírica da judicialização da saúde, que é fundamental para elaboração de políticas públicas. Enquanto as plataformas eletrônicas de base de dados do Poder Judiciário não forem oferecidas em caráter unificado, de modo igualitário, a virtualização dos processos não conseguirá garantir a ampliação do acesso à informação da Justiça, pelo contrário, poderá intensificar a disparidade existente entre o acesso público e privado à Justiça.

O futuro há de ser o processo digital e para que seja de modo eficaz, deve ser cautelosamente instituído, com análise de resultados, falhas e melhoramentos, adaptando os operadores e a sociedade como um todo. Abre-se um enorme potencial para análise e qualificação das políticas públicas, não apenas vinculadas ao setor saúde. Ressalta-se que esse movimento demanda políticas sociais estratégicas que promovam acesso irrestrito da sociedade e pesquisadores (as) em saúde, dentro dos limites das leis vigentes, inclusive com base em dados de fácil acesso em seus sítios eletrônicos.

Portanto, reforça-se a necessidade de aperfeiçoamento estratégico no sentido de padronizar os sistemas eletrônicos usados pelo Poder Judiciário para reger processos judiciais e pesquisas empíricas em saúde, haja vista que a configuração adotada, limita e dificulta pesquisas e análises que podem inclusive nortear a criação de políticas públicas voltadas para a identificação e controle do fenômeno da judicialização da saúde.
